# Prospecting Endophytic Bacteria Endowed With Plant Growth Promoting Potential Isolated From *Camellia sinensis*

**DOI:** 10.3389/fmicb.2021.738058

**Published:** 2021-09-30

**Authors:** Shabiha Nudrat Hazarika, Kangkon Saikia, Atlanta Borah, Debajit Thakur

**Affiliations:** ^1^Microbial Biotechnology Laboratory, Life Sciences Division, Institute of Advanced Study in Science and Technology, Guwahati, India; ^2^Department of Molecular Biology and Biotechnology, Cotton University, Guwahati, India

**Keywords:** endophytic bacteria, *Camellia sinensis*, plant growth promotion, bio-inoculum, 16S rRNA gene, diversity, biofilm

## Abstract

Endophytes are well-acknowledged inoculants to promote plant growth, and extensive research has been done in different plants. However, there is a lacuna about the endophytes associated with tea clones and their benefit to promote plant growth. The present study focuses on isolating and characterizing the beneficial endophytic bacteria (EnB) prevalent in commercially important tea clones cultivated in North Eastern India as plant growth promoters. Diversity of culturable EnB microbiome, *in vitro* traits for plant growth promotion (PGP), and applicability of potent isolates as bioinoculant for *in vivo* PGP abilities have been assessed in the present study. A total of 106 EnB identified as members of phyla Proteobacteria, Firmicutes, and Actinobacteria were related to 22 different genera and six major clusters. Regarding PGP traits, the percentage of isolates positive for the production of indole acetic acid, phosphate solubilization, nitrogen fixation siderophore, ammonia, and 1-aminocyclopropane-1-carboxylic acid deaminase production were 86.8, 28.3, 78.3, 30.2, 95.3, and 87.7, respectively. In total, 34.0, 52.8, and 17.0% of EnB showed notable production of hydrolytic enzymes like cellulase, protease, and amylase, respectively. Additionally, based on the bonitur score, the top two isolates K96 identified as *Stenotrophomonas* sp. and M45 identified as *Pseudomonas* sp. were evaluated for biofilm formation, motility, and *in vivo* plant growth promoting activity. Results suggested strong biofilm formation and motility in K96 and M45 which may attribute to the colonization of the strains in the plants. Further *in vivo* plant growth promotion experiment suggested sturdy efficacy of the K96 and M45 as plant growth promoters in nursery condition in commercial tea clones Tocklai vegetative (TV) TV22 and TV26. Thus, this study emphasizes the opportunity of commercialization of the selected isolates for sustainable development of tea and other crops.

## Introduction

Plants interact with a broad range of diverse bacteria having a beneficial, neutral, or pathogenic impact on their hosts, in both natural and managed ecosystems. The majority of bacterial symbionts known as epiphytes colonize the rhizosphere (Compant et al., 2005; Lugtenberg and Kamilova, 2009). But a subset of bacteria from the rhizosphere are capable to enter and proliferate within plants as endophytes establishing a mutualistic association with the plants (Rosenblueth and Martínez-romero, 2006; Hallmann and Berg, 2007). Endophytes (bacteria or fungi) are an endosymbiotic group of microorganisms, ubiquitous in nature and well-known to dwell inside the plant endosphere without causing any apparent harmful effect on the host plant (Hallmann et al., 1997). Endophytic bacteria (EnB) have been isolated from almost all plants and plant parts such as roots, stems, leaves, barks, floral organs, and even seeds (Ryan et al., 2008; Compant et al., 2011; Dudeja et al., 2012). EnB studies in rice (Fujita et al., 2017), wheat (Emami et al., 2019), tomato (Khan et al., 2014), potato (Sessitsch et al., 2004), sugarcane (Mendes et al., 2007), and many other crops, medicinal plants, halophytes, and woody trees were mainly focussed, but endophytes from tea plants were rarely considered. To maintain stable symbiosis, endophytes ameliorate the host plant to tolerate abiotic and biotic stresses through phosphate solubilization (Liu et al., 2014; Oteino et al., 2015), nitrogen fixation (Hurek and Reinhold-Hurek, 2003; Zhu et al., 2012), indole-3-acetic acid (IAA) (Patten and Glick, 1996; Hassan, 2017), siderophore (Zhang et al., 2011), and 1-aminocyclopropane-1-carboxylic acid (ACC) deaminase (Zhang et al., 2011; Khan et al., 2014) synthesis and augment the resistance of plants to insects, pests, and pathogens by producing bioactive metabolites (Etminani and Harighi, 2018) and polymer hydrolyzing enzymes such as cellulase, amylase, chitinase, and protease (El-Deeb et al., 2012) and promote plant growth and development. Bacterial endophytes that have potentially beneficial effects on plant growth and development has been found in many genera including *Arthrobacter, Azoarcus, Azospirillum, Azotobacter, Bacillus, Burkholderia, Curtobacterium, Erwinia, Gluconobacter, Klebsiella, Serratia, Pantoea, Herbaspirillum, Rahnella, Pseudomonas*, and *Xanthomonas* (Zinniel et al., 2002; Berg et al., 2005; Compant et al., 2011).

*Camellia sinensis* (L) O. Kuntze (Tea), an economically important crop, is the world’s most-consumed non-alcoholic beverage which has been commercially produced by more than 30 countries worldwide (Hayat et al., 2015), with 1283.3 M. Kgs produced by India making it the world’s second-largest producer of tea (Sarani, 2020). In India, tea is extensively cultivated in Assam, Darjeeling, Nilgiri, and other North-Eastern (NE) states of India (Mondal et al., 2004). Assam plays a pivotal role in the national economy by producing 623.23 M. Kgs of tea, being the leading producer and exporter of commercial tea (Sarani, 2020). With the favorable climatic and topographic conditions of Meghalaya, suitable tea varieties brought from Assam and Darjeeling were transplanted at tea gardens located at Umsning, Meghalaya, India, in 1978 which were acclaimed by tea makers to be of very high quality (Directorate of Food Processing Meghalaya, n.d.). Tocklai Tea Research Institute (TTRI), Jorhat, Assam, India, has released 33 Tocklai Vegetative (TV) series tea clones, 153 region-specific garden series clones, and 16 biclonal seed stocks to the tea industry (Patel et al., 2019) and classified them based on their yield and quality into a standard clone (greater than average quality and yield), quality clone (high quality with average yield), and yield clone (average quality with high yield) (Das et al., 2012).

Owing to the production of piles of nutraceuticals in tea plants, consumption of tea drinks is related to numerous medicinal and nutritional benefits (Sharangi, 2009) such as antioxidant (Frei and Higdon, 2003), anti-inflammatory (Hsu et al., 2003), anti-carcinogenic (Kris-Etherton et al., 2002; Cooper et al., 2005), anti-allergic (Maeda-Yamamoto et al., 2007), and anti-microbial effect (Hamilton-Miller, 1995; Siddiqui et al., 2016). Although studies on tea phytochemicals and their potent effects have been well characterized, research on endophytic bacteria associated with tea clones is rather sparse.

Compared to the rhizospheric bacterial community (Dutta and Thakur, 2017) and endophytic fungal community (Win et al., 2018), EnB community of tea plants has received less attention. The interaction studies between EnB microflora associated with different tea clones cultivated especially in the NE region of India have not yet been investigated thoroughly. The culturable microbial diversity of EnB associated with tea clones has barely been explored. Also, the ability to utilize the EnB as plant growth promoters has not been well reported. Thus, the current study aims to investigate EnB community prevalent in selected commercially popular tea clones (TV1, TV22, TV9, Teenali 17, and TV25) using molecular, phylogenetic, and functional approaches. To evaluate the plant growth stimulation by plant growth-promoting bacteria (PGPB), phosphate solubilization, production of phytohormone, siderophore and ammonia, biological nitrogen fixation, and stress alleviation by production of ACC deaminase were carried out. To identify and explore the microbial diversity of the endophytic isolates molecular techniques such as 16S rRNA and phylogenetic analysis were used. Further, to determine the efficacy of the potent plant growth-promoting isolates, a nursery experiment was performed using two different yield clones, TV22 and TV26, which are extensively used for commercial cultivation in NE India. Thus, the results endowed the importance of EnB microflora as plant growth promoters that can be used to develop microbial formulations to reduce the use of agrochemicals and for sustainable development of tea plants including the soil.

## Materials and Methods

### Sampling of Plant Material

Random sampling method was used to collect leaf and root samples from selected healthy tea plants of five clones, TV1, TV9, Teenali 17, TV22, and TV25 (Table 1), and all clones were selected based on their commercial importance. The samples were collected from two locations: Kopati Tea estate, Assam, India, and MEG tea estate, Meghalaya, India. The leaves and roots were collected aseptically in sterile ziplock bags as composite samples for each tea clone, transported to the laboratory in an ice box, and further processing for isolation was done within 1 month.

**TABLE 1:** Collection of Tea clones.

Sl. no.	Collected samples			Tissue of origin	Sampling site and GIS location
	Tea clones	Jat/Population/Parents	Type of clone		
1	TV1	Assam-China (Cinnamara)	Standard	Leaf, root	Kopati tea estate, Darrang, Assam, India (26.5875°N, 92.2507°E); Meg Tea, Tea Development Centre, Umsning, Meghalaya, India (25.741729° N, 91.889094° E)
2	TV9	Burma (Cambod)	Standard	Leaf, root	
3	TV22	Cambod type (Indo-China)	Yield	Leaf, root	
4	Teenali 17	Cambod type	Estate	Leaf, root	
5	TV25	Cambod type: Ayapathar D × Ayapathar A (DA/4)	Yield	Leaf, root	

### Isolation of Endophytic Bacteria From Tea Clones

EnB was isolated by using surface sterilization and serial dilution method as described (Schulz et al., 1993; Sturz et al., 1998) with slight modifications. In brief, plant materials were washed for 3–5 min in running tap water to remove any debris and rinsed in 70% ethanol for 30 s followed by surface sterilization for 5 min using 1.5 and 2% v/v sodium hypochlorite solution for leaves and roots, respectively, followed by washing with sterile distilled water thrice for 5 min. The efficacy of surface sterilization was validated by spreading 100 μL aliquots of distilled water on nutrient agar (NA) plates taken from the last wash of the samples.

The surface-sterilized samples were excised and macerated in 0.9% sodium chloride (NaCl) with a sterile mortar pestle. EnB isolation was done by spread plate method, 100 μL of the aliquots were spread on selective media, namely Nutrient Agar, King’s B agar, Pikovskaya’s agar, Pseudomonas agar (HiMedia, India) followed by incubation at 28 ± 2°C for 48–72 h. Distinguished colonies based on phenotypic characters (size, elevation, color, form, and margin) were selected and pure culture of the isolates were made to determine the culturable bacterial population. For further studies and long-term preservation pure cultures were maintained at 4°C and −80°C as 20% (w/v) glycerol stocks, respectively.

### Molecular Characterization of Endophytic Bacteria

#### DNA Extraction and 16S rRNA Gene Amplification

Genomic DNA was extracted from isolates according to the protocol in the maker’s instructions as mentioned in QIAamp DNA mini kit (Qiagen, Hilden, Germany). The purity and concentration of the eluted DNA were quantified by NanoDrop spectrophotometer (Thermo Fisher Scientific, United States) and stored at 4°C until required for PCR.

In addition, 16S rRNA gene of the isolates was amplified using 1 μL (10 μmol L^–1^) forward (27F 5′-AGA GTT TGA TCC TGG CTC AG-3′) and reverse primers (1492R 5′- GGT TAC CTT GTT ACG ACT T-3′), 5 μL (10x) Taq buffer, 2.5 μL (2.5 mmol L^–1^) dNTPs, 0.5 μL (2.5 U) Taq polymerase in a total volume of 50 μL reaction mixture. PCR cycling conditions were set up in a Thermal cycler (Eppendorf, United States) as follows: 94°C for 5 min for initial denaturation of DNA, followed by 35 cycles of denaturation at 94°C for 30 s, annealing of primer at 55°C for 40 s, extension of DNA strand at 72°C for 1 min and final extension at 72°C for 10 min (Passari et al., 2015). The amplicons were then separated on a 1.2% agarose gel with ethidium bromide, visualized and documented in a BioRad Gel Doc XRS+ (Hercules, CA, United States). The PCR amplicons were then outsourced to 1st Base DNA Sequencing service, Malaysia for Sanger sequencing to determine the nucleotide sequence of DNA.

#### Sequence Analysis and Phylogenetic Analysis

The 16S rRNA sequences were subjected to de-novo assembly using UGENE 37.0 and contigs generated were queried for homology using NCBI BLASTn and EZ taxon server 2.1 programs. The identified accessions were then submitted to GenBank and accession numbers were collected for each isolate. The multiple sequence alignment was carried out using MAFFT v7.45 (Katoh and Standley, 2013) and corrected by trimAl v1.4 using the gappyout algorithm (Capella-Gutiérrez et al., 2009) followed by a validation step using pmodel test tool of ETE3 toolkit (Huerta-Cepas et al., 2016). The evolutionary relationship between the accessions was analyzed by the maximum likelihood method using RAxML with 1,000 bootstrap steps (Stamatakis, 2014) and a phylogenetic tree was constructed using. Fig Tree 1.4.4 (Rambaut, 2018).

### Screening of Plant Growth Promotion Traits *in vitro*

The EnB isolates were screened *in vitro* for identifying the traits required for nutrient acquisition and plant growth, like production of phytohormone, ammonia, and iron chelating agents, fixation of nitrogen from the atmosphere, production of stress alleviating enzymes, and hydrolytic enzymes. For all the PGP assays *Bacillus pseudomycoides* strain SN29 (GenBank accession no. KJ767523) (Dutta et al., 2015) was used as a positive control.

#### Estimation of Production of Phytohormone Indole-3-Acetic Acid

Gordon and Weber’s (1951) method was used to determine IAA production quantitatively and qualitatively with modifications. EnB isolates were inoculated in minimal salt (MS) medium amended with 2 mg mL^–1^ L-Tryptophan and incubated in an orbital shaker (170 rpm) at 28 ± 2°C for 48 h. To obtain bacterial cells free supernatant centrifugation at 10,000 rpm for 15 min was done. To the supernatant Salwoski reagent (0.5 M FeCl_3_ and 70% perchloric acid) was added in 1:2 ratio (v/v) and incubated in dark for 25 min at room temperature. After incubation optical density was measured at 530 nm using a multimode reader (Varioskan, Thermo Fisher Scientific). Commercial IAA (Sigma-Aldrich) was used as a standard to quantify IAA production. The experiment was done in triplicates.

#### Estimation of Phosphate Solubilization

Phosphate solubilization by EnB isolates was screened by spot inoculation on Pikovskaya’s agar medium (PKA) followed by incubation at 28 ± 2°C for 48 h. To quantify the potential of isolates to solubilize tricalcium phosphate, they were grown in NBRIP (National Botanical Research Institute’s Phosphate Growth) medium at 28°C for 48 h as described by Fiske and Subbarow (1925) and centrifuged at 10,000 rpm for 20 min to obtain supernatant. The amount of phosphate content in the supernatant was determined by adding ammonium molybdate reagent in equal volume and measured by colorimetry at 650 nm. A standard curve of KH_2_PO_4_ was used to determine the amount of phosphate solubilized which was expressed in mg L^–1^. The experiment was done in triplicates.

#### Estimation of Siderophore Production

Quantitative analysis of siderophore production was determined by Chrome Azurol S (CAS) shuttle assay as described (Schwyn and Neilands, 1987; Alexander and Zuberer, 1991). The EnB isolates were grown in MS media at 28 ± 2°C for 48 h and after incubation culture was centrifuged at 10,000 rpm for 10 min and mixed with an equal volume of CAS reagent. The un-inoculated MS medium served as a control. The colorimetric measurements were performed by using a multimode reader at 630 nm. The percentage of siderophore produced (psu) by isolates was calculated by the following formula as described (Payne, 1994).


$$Siderophoreproductionunits\left(psu\right)=\frac{\left(Ar-As\right)x100}{Ar}$$

where *Ar* = absorbance of reference and *As* = absorbance of the sample at 630 nm

#### Estimation of Ammonia Production and Nitrogen Fixation

Ammonia production was determined as described (Yuen and Pollard, 1954; Singh et al., 2014). EnB isolates were inoculated into Peptone Water Broth medium and incubated at 28 ± 2°C, 150 rpm for 48 h. The bacteria cells were removed by centrifugation at 8,000 rpm for 5 min. To the supernatant, Nessler’s reagent was added in a 2:1 ratio (v/v). Yellow to brown color development indicated ammonia production. Ammonium sulfate (0.1–5.0 μmol mL^–1^) was used as a standard to quantify the amount of ammonia production, the absorbance of which was measured at 450 nm by a multimode reader and expressed as μmol mL^–1^. The experiment was done in triplicates.

Nitrogen-fixing abilities of the EnB isolates were determined qualitatively by culturing in Nitrogen-free (NF) medium containing (g L^–1^) mannitol 20 g, K_2_HPO_4_ 0.2 g, NaCl 0.2 g, MgSO_4_.7H_2_O 0.2 g, K_2_SO_4_ 0.1 g, CaCO_3_ 5 g, and agar 20 g and Jensen’s agar medium by spot inoculation method as described (Kifle and Laing, 2016). The growth of the isolates was monitored after incubation at 28 ± 2°C for 48 h.

#### Qualitative Estimation of 1-Aminocyclopropane-1-Carboxylic Acid Deaminase Activity

ACC deaminase activity of the EnB isolates was qualitatively assessed by spot inoculation method in Dworkin and Foster (DF) salt medium (Dworkin and Foster, 1958) amended with 3 mM ACC as sole nitrogen source. The inoculated plates were incubated for 48 h at 28 ± 2°C and monitored. The colonies growing on the plates were recognized as ACC deaminase producers (Glick et al., 2007).

### Screening of Hydrolytic Enzyme Production

For the production of carboxymethyl cellulase (CMCase), EnB isolates were spot inoculated on M9-CMC agar plates containing (g L^–1^), NaNO_3_ 1 g, K_2_HPO_4_ 1 g, KCl 1 g, MgSO_4_ 0.5 g, yeast extract 5 g, agar 15 g, and CMC 2 g as substrate and incubated for 48 h at 28 ± 2°C. The incubated plates were flooded with 0.2% congo red solution for 15 min and de-stained with 0.1 M NaCl for 15 min. The formation of clear halo zones around colonies after 3–5 min confirmed cellulose hydrolysis (Meddeb-mouelhi et al., 2014).

The starch degrading ability of isolates to produce amylolytic enzymes was determined by spot inoculation on starch agar plates containing (g L^–1^) meat extract 3 g, peptic digest of animal tissue 5 g, starch soluble 2 g, and agar 15 g. Lugol solution (5 g iodine crystals and 10 g potassium iodide in 100 mL of distilled water) was flooded in plates for 2 min after incubation at 28 ± 2°C for 48 h. On starch hydrolysis, a clear zone around the colonies indicated amylase production (Iverson and Millis, 1974).

Protease production by EnB isolates was evaluated by spot inoculation on skim milk agar (SMA) medium (Sgroy et al., 2009). The inoculated plates were incubated at 28 ± 2°C for 48 h. Proteolytic activity was confirmed by the formation of clear halo zones around colonies.

### Quantification and Microscopic Evaluation of Bacterial Biofilm Development

*In vitro* biofilm formation was quantified using microtiter plate assay method described (O’Toole and Kolter, 1998) with minor modifications. Overnight grown cultures of EnB were re-suspended in nutrient broth (NB) medium and incubated in a rotary shaker (180 rpm) at 28°C to obtain the final OD_600_ = 0.2 (10^7^ CFU mL^–1^) bacterial cells. Aliquots of 200 μL of EnB cultures were moved to 96 well microtiter plate and incubated at 28 ± 2°C for 48 h in static condition. NB broth without inoculation was taken as control. To remove the planktonic bacteria, the wells were emptied and washed thrice with 1X phosphate-buffered saline (PBS), pH 7.2 followed by staining with 0.1% crystal violet solution for 30 min. Excess bound stains on wells were removed and the wells were washed with 1X PBS and allowed to air dry. The bound crystal violet was then solubilized using 100% ethanol and the intensity of biofilm formation was measured at 570 nm using a multimode plate reader (Gupta et al., 2019).

Light microscopy and confocal microscopy were used to evaluate bacterial biofilm development. Freshly grown overnight bacterial cultures (20 μL) of K96 and M45 were inoculated in sterile glass coverslip (20 mm) and placed in a 60 mm culture plate and incubated at 28 ± 2°C for 24–48 h under static condition. Loosely adhered cells were removed from the surface by washing glass coverslip thrice with 1X PBS and stained with 0.1% crystal violet (CV) for 10 min and 0.1% acridine orange (AO) for 20 min followed by removal of excess stain by washing with 1X PBS and allowed to dry. To examine the extent of microbial attachment or biofilm formation on the glass surface the slides stained with CV were visualized under a compound microscope.

Confocal Laser Scanning Microscope (Clsm) imaging was performed using a Leica Tcs Sp8 confocal laser scanning microscope (Leica, Mannheim, Germany) equipped with a visible light laser, Leica Dmi 6000B inverted light microscope, and an Hc Pl Apo Cs2 63X/1.40 Oil immersion objective. Acridine Orange (Ao) was excited using a 488 nm argon laser line, while the fluorescent emission was detected from 502 to 526 nm. The pinhole aperture size was adjusted to 1.8 Airy units and detector gain was set to 630 for visualization of the biofilm. In the z-plane, 36 fluorescent optical images were collected to span the full depth of the biofilms. Leica Application Suite X software was used for visualization and ImageJ software was used for the analysis of the images.

### Cell Surface Hydrophobicity Assay

Microbial Adhesion to Hydrocarbons (MATH) method was used to establish CSH by K96 and M45 EnB isolates as described (Rosenberg, 2006). The percentage of hydrophobicity was described as follows:


$$Percentageofhydrophobicity\left(\%\right)=\frac{\left(A0-A1\right)x100}{A0}$$

where A0 is the initial absorbance of bacterial culture at 400 nm and A1 is the final absorbance of the aqueous phase at 400 nm.

### Motility Assay

Bacterial motility assays (swimming and swarming) were performed by the protocol described (Gupta et al., 2019) with slight modification. Luria broth (LB) medium with 0.3% agar (w/v) and 0.5% (w/v) agar supplemented with 5 g L^–1^ D-glucose was used for swimming motility and swarming motility assay, respectively. To study the bacterial migration freshly grown bacterial culture (5 μL, OD_600_ = 0.2) of cell density (10^7^ cells mL^–1^) was inoculated on each assay plate and incubated in an inverted position at 28 ± 2°C for 48 h and the swarm diameter for both swimming and swarming motilities was measured at 24 and 48 h of incubation and expressed in mm. The twitching motility assay was done by inoculation of freshly grown bacterial isolates in LB medium with 0.6% agar followed by incubation at 28 ± 2°C for 48 h (Henrichsen, 1983). The bacterial twitching motility was visualized under the microscope (Motic BA410).

### Plant Growth Promotion Experiment

#### Plant Material

For the plant growth promotion experiment in nursery conditions, 3-months-old tea vegetative clones TV22 (Indo-China) and TV26 (Ayapthar D × Ayapathar A) were collected from Bateli Tea estate, Assam. These TV clones are Cambod type yield clones (high yield average quality). The procured tea clones were propagated mainly through vegetative propagation using cuttings for commercial cultivation. The cuttings consist of a 3–4 cm long single leaf with a stem below the node about 2.5 cm and about 0.5 cm of stem above it. Before propagation, the cuttings were first treated with 0.1% solution of zinc sulfate. For callusing or rooting of cuttings they are transferred to nursery beds. After rooting (6–8 weeks), the cuttings were transferred to polythene sleeves where they were grown and maintained in nursery condition. For vegetative propagation of tea in the tea estate, the sleeves were prepared following standard protocol described in Field Management Notes, TTRI, Jorhat. The polythene sleeves prepared are 15 ± 17.7 cm layflat wide, 20 ± 25 cm long, and 150 gauge thick in size. The soil used in the sleeves contains well-drained sandy loam (15–20% clay less than 0.002 mm, 0–50% silt 0.002–0.2 mm, and 50–70% sand greater than 0.2 mm) of good tilth having ideal pH of 4.5.

#### Preparation of Bacterial Inoculum and Treatment

For the PGP experiment in nursery condition, two potent isolates K96 (*Stenotrophomonas* sp., GenBank Accession number MW905624) and M45 (*Pseudomonas* sp., GenBank Accession number MZ008002) were selected based on *in vitro* PGP abilities. For mass production, the pure bacterial cultures were inoculated in NB (HiMedia, India) and grown for 24 h at 28 ± 2°C at 180 rpm. The cultures were centrifuged at 10,000 rpm for 15 min and the pellets were washed twice with PBS to obtain a final suspension of 10^8^ CFU mL^–1^ (Xu et al., 2014). Then, 10 mL of the resulting cell suspension was used to treat the tea clones in the nursery condition by soil drenching method in 15 days interval for 6 months (Dutta and Thakur, 2017; Borah et al., 2019). The nursery experiment was divided into three treatments, namely T1:K96 inoculation, T2 :M45 inoculation, T3 :inoculated with consortia of K96 and M45, and control, without bacterial inoculum, with three replicates each. To retain the soil moisture the saplings were watered once daily until harvesting. Harvesting of the tea saplings was done after 6 months of the last inoculum treatment. Physiological parameters of plants (number of leaves, shoot and root length along with their fresh and dry weight, and chlorophyll content) were recorded and compared to control.

#### Chlorophyll Estimation

Mature tea leaves were obtained from the treated and untreated tea clones to quantitatively determine the chlorophyll content in the leaf tissues as described by Hiscox and Israelstam (1979). To facilitate the extraction of chlorophyll, 100 mg of fresh leaf tissue was cut into small pieces and immersed in 7 mL DMSO (HiMedia). After extraction at 65°C for 30 min in dark using a water bath, the supernatant was decanted and absorbance was determined at 645 and 663 nm against DMSO as blank using a multimode reader. Chlorophyll a and b were calculated by the following formula (Arnon, 1949):


$$Chlorophylla\left(mg/g\right)=12.7\left(A663\right)-2.69\left(A645\right)x\frac{V}{1000xW}$$

$$Chlorophyllb\left(mg/g\right)=22.9\left(A645\right)-4.68\left(A663\right)x\frac{V}{1000xW}$$

where A = absorbance at specific wavelengths; V = final volume of chlorophyll extract; W = fresh weight of tissue extracted.

#### Data Analysis

The Vennture software (Martin et al., 2012) was used to make a Venn diagram to represent all the isolates producing different PGP traits. The plant growth parameters were analyzed statistically by subjecting to two-way Anova (analysis of variance). Statistically significant data was determined at *p* ≤ 0.05. The interrelationships among the treatments and vegetative parameters were evaluated using principal component analysis (PCA) in Matlab R (2017a). In addition, to determine the absolute value difference between group means of control and treatment fold change analysis was done. The resulting values are generated in a log2 scale such that the up or down-regulation will be equidistant to the baseline.

## Results

### Endophytic Bacteria Isolation From Selected Tea Clones

**FIGURE 1:**
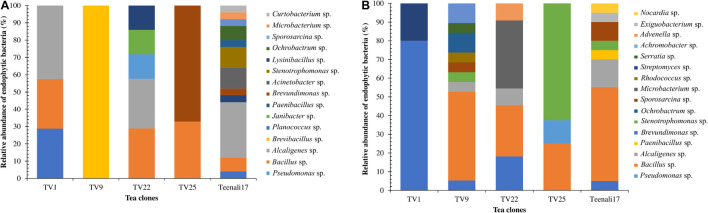
Relative abundance (in percentage) of endophytic bacterial genera procured from **(A)** leaf and **(B)** roots of five different tea clones (TV1, TV9, TV22, TV25, and Teenali 17).

Altogether 106 culturable EnB were isolated from leaves and roots of five different tea clones using different enriched media and identified based on their morphological characteristics. The population density of EnB isolates in the selected tea clones varied from 3.2 to 4.4 log_10_ colony forming units g^–1^ (CFU g^–1^) per fresh weight (Supplementary Figure 1). In addition, 59.43% EnB (*n* = 63) isolates were obtained from roots and 40.56% (*n* = 43) from leaves. The bacterial isolates were confirmed to be endophytes as no bacterial colonies were observed in the control plates. The relative abundance of each EnB isolated was determined at the genus level in both leaf and root samples of the five different tea clones. In leaf samples, as evident from Figure 1A, *Alcaligenes* sp. (42.58%) was most abundant in TV1 clone, *Brevibacillus* sp. (100%) in TV9, *Bacillus* sp. (28.83%) and *Alcaligenes* sp. (28.83%) in TV22, *Brevundimonas* sp. (67.14%) in TV25, and *Alcaligenes* sp. (32.05%) in Teenali17. In root samples, as shown in Figure 1B, *Pseudomonas* sp. (79.97%) was most abundant in TV1, *Bacillus* sp. (47.38%) in TV9, *Microbacterium* sp. (36.67%) in TV22, *Stenotrophomonas* sp. (62.52%) in TV25, and *Bacillus* sp. (49.98%) in Teenali 17.

The Shannon diversity indices of EnB were more in leaf (H = 2.3) as compared to the root (H = 2.14). Simpson diversity index was 0.125 in leaf and 0.177 in the root. Species richness (S) was found to be 63 in root and 43 in leaf samples. The species evenness value was found to be 0.610 in leaf and 0.516 in the root. The average population size is found to be 2.87 and 3.94 in leaf and root samples, respectively. The population of EnB isolates with their diversity indices is shown in Table 2.

**TABLE 2:** Population of endophytic bacterial isolates with diversity indices.

Isolate name	Leaf	Root
*Acinetobacter* sp.	3	0
*Stenotrophomonas* sp.	3	7
*Brevundimonas* sp.	3	1
*Lysinibacillus* sp.	1	0
*Pseudomonas* sp.	3	8
*Ochrobactrum* sp.	2	1
*Alcaligenes* sp.	13	5
*Sporosarcina* sp.	1	3
*Paenibacillus* sp.	2	1
*Bacillus* sp.	7	24
*Rhodococcus* sp.	0	1
*Exiguobacterium* sp.	0	1
*Serratia* sp.	0	1
*Achromobacter* sp.	0	2
*Planococcus* sp.	1	0
*Janibacter* sp.	1	0
*Brevibacillus* sp.	1	0
*Curtobacterium* sp.	1	0
*Advenella* sp.	0	1
*Streptomyces* sp.	0	2
*Nocardia* sp.	0	1
*Microbacterium* sp.	1	4
Taxa	15	16
Total number of organisms	43	63
Shannon diversity index_H	2.3	2.14
Simpson diversity index_SDI	0.125	0.177
Species evenness	0.62	0.516
Berger-Parker Dominance Index	0.302	0.381
Margalef Richness Index	3.72	3.62
Menhinick Index	2.29	2.02

### Molecular Identification of Endophytic Bacteria Isolates

**FIGURE 2:**
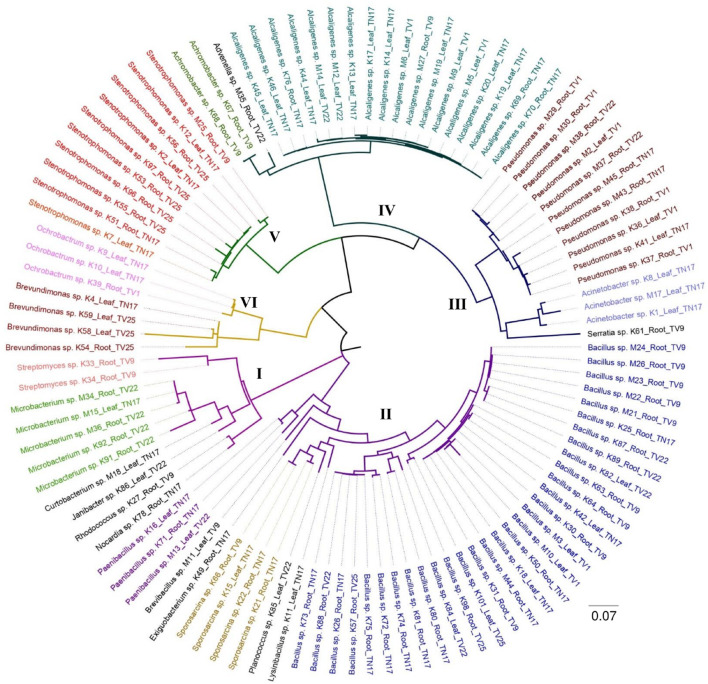
The maximum likelihood phylogenetic tree of endophytic bacterial isolates obtained from five different tea clones based on 16S rRNA gene sequences; the scale bar represents the distance from the origin to the most distant taxa. Clades that are distinctly clustered are represented by six different groups.

The present study revealed a diverse population of bacterial isolates within 22 different genera based on 16S rRNA gene sequencing. The sequence results procured from BLAST validated the identity with 91–100% similarity. The 16s rRNA sequence GenBank accession numbers of individual isolates and sample details are listed in Supplementary Table 1. The phylogenetic analysis by maximum likelihood revealed 6 major clusters (Figure 2). Cluster II was the largest consisting of 42 isolates of different genera belonging to *Bacillus* sp., *Sporosarcina* sp., *Paenibacillus* sp., Lysinibacillus sp., *Planococcus* sp., *Brevibacillus* sp., and *Exiguobacterium* sp. Actinobacteria species viz. *Microbacterium* sp., *Streptomyces* sp., *Curtobacterium* sp., *Janibacter* sp*., Rhodococcus* sp*., and Nocardia* sp. were distributed separately in cluster I which consisted of 11 isolates. *Pseudomonas* sp., *Acinetobacter* sp., and *Serratia* sp. were found to be in cluster III containing 15 isolates. Cluster IV comprised of 21 isolates, where it was mostly dominated by *Alcaligenes* sp. followed by *Achromobacter* sp. and *Advenella* sp. All 10 isolates in cluster V belongs to *Stenotrophomonas* sp. and 7 isolates in cluster VI belong to the genera *Ochrobactrum* sp. and *Brevundimonas* sp. Cluster I, II, and the rest of the clusters are found to be divided into two distinct sub-nodes from the origin, following a separate hierarchy. Taxa within the same genera and similar tissue (viz. leaf and root) can be distinguished separately in the subclusters with minimum evolutionary distance. In most of the cases, a diverse phylogenetic relationship was observed between accessions originating from different tea clones.

### Plant Growth-Promoting Characteristics of Bacterial Endophytes

**FIGURE 3:**
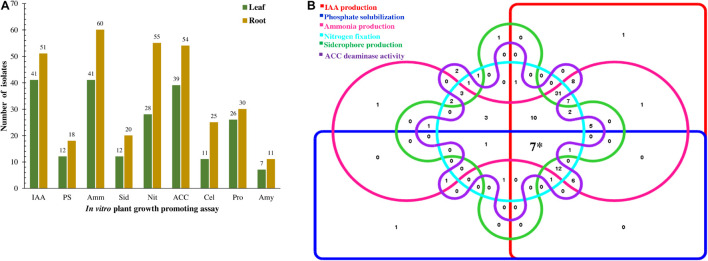
Representation of plant growth-promoting traits showed by endophytic bacteria isolated from the tea clones. **(A)** Quantitative distribution of bacterial isolates according to PGP traits and isolation source and **(B)** Venn diagram illustrating the number of isolates showing PGP traits. The 106 isolates were allocated to 6 different PGP traits. (*7 showed PGP traits for all the attributes).

All the 106 isolates when screened for various PGP traits *in vitro* showed at least one trait as shown in Figure 3A and Supplementary Table 2. On the combined evaluation of all the six PGP traits, seven isolates (K63, K71, K96, M2, M29, M37, and M45) revealed the capacity to produce all the traits (Phosphate solubilization, IAA, Ammonia and Siderophore production, Nitrogen fixation and ACC deaminase activity) which is shown in the Venn diagram in Figure 3B.

#### Indole-3-Acetic Acid Production and Phosphate Solubilization

The ability to biosynthesize IAA was observed in 92 isolates, 41 isolates from leaf, and 51 from the root. The majority of isolates to produce IAA belong to the genera *Bacillus* sp. (26 isolates) followed by *Alcaligenes* sp. (16 isolates), *Pseudomonas* sp. (11 isolates), and *Stenotrophomonas* sp. (10 isolates). Quantitatively IAA production in presence of L-Tryptophan ranges from 3.00 ± 1.19 to 129.84 ± 4.72 μg mL^–1^. Twelve (11.32%) produced more than 40 μg mL^–1^. Isolate *Ochrobactrum* sp. (K39) showed the highest production of IAA of 129.84 ± 4.72 μg mL^–1^ followed by *Stenotrophomonas* sp. (K96) 123.55 ± 2.50 μg mL^–1^.

In addition, 28.30% of all the isolates (*n* = 30), among which 12 from leaf and 18 from roots, were identified to solubilize inorganic phosphate based on the development of clear zones around colonies in Pikovskaya’s agar medium. Among the isolates *Bacillus* sp. (10 isolates) followed by *Pseudomonas* sp. (6 isolates) showed maximum phosphate solubilizing activity. The efficiency of phosphate solubilization varies from 2.79 ± 0.67 to 81.42 ± 5.73 mg L^–1^. The highest phosphate solubilizing ability was shown by *Bacillus* sp. (K98) which solubilized 81.42 ± 5.73 mg L^–1^ calcium phosphate available in the medium followed by *Serratia* sp. (K61) 73.24 ± 0.38 mg L^–1^ and *Stenotrophomonas* sp. (K96) 71.62 ± 2.2 mg L^–1^.

#### Ammonia Production and Nitrogen Fixation

The majority of isolates (101) produced ammonia, accounting for 41 isolates from leaf and 60 isolates from roots. The majority of ammonia producers belong to the genera *Bacillus* sp. (29 isolates) followed by *Alcaligenes* sp. (17 isolates) and *Pseudomonas* sp. (11 isolates). The amount of ammonia produced falls in the range 0.61 ± 0.26–4.92 ± 0.19 μmol mL^–1^ with 39 isolates (36.79%) having the capacity to produce ammonia more than 3.0 μg mL^–1^. *Sporosarcina* sp. (M15) produced the highest amount of ammonia of 4.92 ± 0.19 μmol mL^–1^ in the medium.

Also, 78.30% of the total isolates were capable of growing on NF media and Jensen’s agar media, where 28 were from leaf and 55 from roots. Maximum nitrogen fixation was observed in *Bacillus* sp. (31 isolates) subsequently by *Pseudomonas* sp. (11 isolates), *Stenotrophomonas* sp. (10 isolates), and *Alcaligenes* sp. (10 isolates). NF media and Jensen’s agar media are defined medium without nitrogen used for detection of nitrogen-fixing bacteria. The growth of EnB on medium indicates that these bacteria can fix atmospheric nitrogen.

#### Siderophore Production and 1-Aminocyclopropane-1-Carboxylic Acid Deaminase Activity

Siderophore production in liquid media was detected in 32 (30.18%) isolates among which 12 isolates were from leaf and 20 from roots, where maximum production was shown by genera *Bacillus* sp. (9 isolates). Quantitative estimation of siderophore production showed that isolate *Brevundimonas* sp. (K4) and *Bacillus* sp. (K87) produced the highest quantities of siderophore units (SU) of 83.22% followed by 81.2% SU by *Pseudomonas* sp. (M45) and 79.06% SU by *Stenotrophomonas* sp. (K96). A total of 17 isolates produced significantly higher quantities of siderophores above 40% siderophore units.

Out of 93 EnB isolates, 39 from leaf and 54 from roots utilized ACC as the sole nitrogen source and exhibit ACC deaminase activity. Genera *Bacillus* sp. (29 isolates) showed maximum ACC deaminase activity followed by *Alcaligenes* sp. (15 isolates) and *Stenotrophomonas* sp. (10 isolates). The isolates showing similar diameters as the control plate was considered to be weak or negative isolates and the isolates showing greater diameter than control plates were considered to be positive.

### Hydrolytic Enzyme Production Assays

Qualitative analysis of 106 EnB isolates for hydrolysis of extracellular enzymes such as cellulase, protease, and amylase was performed. A total of 56 (52.83%) isolates hydrolyzed proteins by the formation of clear zones around colonies in SMA medium. For amylase screening, 20 (18.87%) isolates hydrolyzed starch by the production of amylase in starch agar medium by the formation of clear zones around bacterial isolates and cellulase enzyme production by 37 (34.90%) isolates were confirmed by the development of clear halo zones in the vicinity of colonies in M9-CMC medium (Figure 3A, Table 3, and Supplementary Table 2).

**TABLE 3:** Endophytic bacterial isolates with their PGP traits and general ranking for their ability to function as plant growth promoters based on bonitur scale.

Isolate code	Organism name	Accession no.	Plant growth promoting traits						Hydrolytic enzymes			Total assessment points (13)	Rank
						
			IAA^a^	PS^b^	Amm^c^	Sid^d^	Nit^e^	ACC^f^	Cel^g^	Pro^h^	Amy^i^		
K96	*Stenotrophomonas* sp.	MW905624	3	3	1	1	1	1	1	1	1	13	1st
M45	*Pseudomonas* sp.	MZ008002	2	2	1	1	1	1	1	1	1	11	2nd
K55	*Stenotrophomonas* sp.	MW898448	2	1	1	0	1	1	1	1	1	9	3rd
K61	*Serratia* sp.	MN493913.1	1	3	1	0	1	1	1	1	0	9	4th
K98	*Bacillus* sp.	MN577376.1	0	3	1	1	1	1	0	1	1	9	5th
K9	*Ochrobactrum* sp.	MN493877.1	1	0	1	1	1	1	1	1	1	8	6th
K51	*Stenotrophomonas* sp.	MN493906.1	1	0	1	1	1	1	1	1	1	8	7th
K71	*Paenibacillus* sp.	MN493921.1	1	2	1	1	1	1	0	1	0	8	8th
M2	*Pseudomonas* sp.	MW905608	1	1	1	1	1	1	1	1	0	8	9th
M3	*Bacillus sp.*	MN577378.1	1	3	1	0	1	1	0	1	0	8	10th

*IAA^a^, Indole Acetic Acid production (1 ≤ 50, 2 = 50–100, 3 ≥ 100 μg mL^–1^); PS^b^, Phosphate solubilization (1 ≤ 30, 2 = 30–60, 3 ≥ 60 μg mL^–1^); Amm^c^, Ammonia production (μmol mL^–1^) (1 = positive); Sid^d^, Siderophore production (%) (1 = positive); Nit^e^, Nitrogen fixation (1 = positive); ACC^f^, ACC deaminase activity (1 = positive); Cel^g^, Cellulase activity (1 = positive); Pro^h^, Protease activity (1 = positive); Amy^i^, Amylase activity (1 = positive). 0 indicates no activity or growth.*

### Concluding Evaluation of the *in vitro* Plant Growth Promotion Traits

A bonitur scale was generated similar to that described by El-Sayed et al. (2014) to select the best EnB isolates having high plant growth-promoting potential. In this scale PGP traits examined are scored. The maximum possible score in the traits examined here is 13 points: 3 points each for IAA production and phosphate solubilization; 1 point each for nitrogen fixation, ammonia, siderophore, and ACC deaminase production, and production of hydrolytic enzymes was given as the assessment values. The assessment revealed that isolate *Stenotrophomonas* sp. (K96) was the foremost isolate exhibiting the highest score of 13 points followed by *Pseudomonas* sp. (M45) exhibiting 11 points. Based on the bonitur scale the top 10 EnB isolates with the ranking for their ability to function as plant growth promoters are shown in Table 3 and the ranking of all the isolates are shown in Supplementary Table 2.

### Biofilm Formation Assay

**FIGURE 4:**
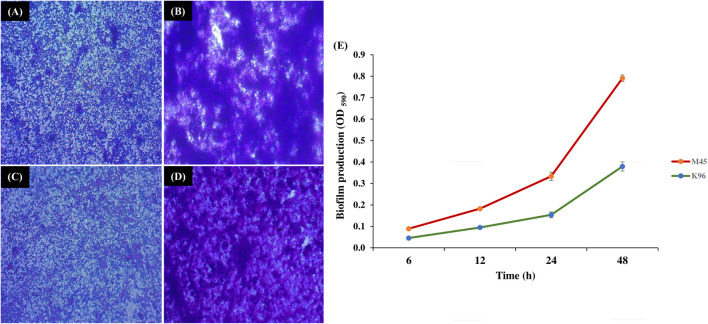
Biofilm formation by potent bacterial isolates. Light microscopy images of biofilm formation by **(A,B)** M45 (*Pseudomonas* sp.), and **(C,D)** K96 (*Stenotrophomonas* sp.) at 24 and 48 h, respectively. The biofilms were stained by crystal violet; **(E)** Quantification of biofilm production for 48 h by M45 and K96 after elution of crystal violet stain by ethanol.

Bacterial isolate M45 showed strong biofilm formation and isolate K96 showed moderate biofilm formation at 48 h, i.e., during the stationary growth phase. The same was observed when biofilm was developed on a glass slide and observed under a compound microscope at 24 and 48 h after staining with crystal violet (Figures 4A–D). The attachment of bacterial cells increased significantly after 12 h (initial period of lag phase) as assessed using crystal violet (Figure 4E).

**FIGURE 5:**
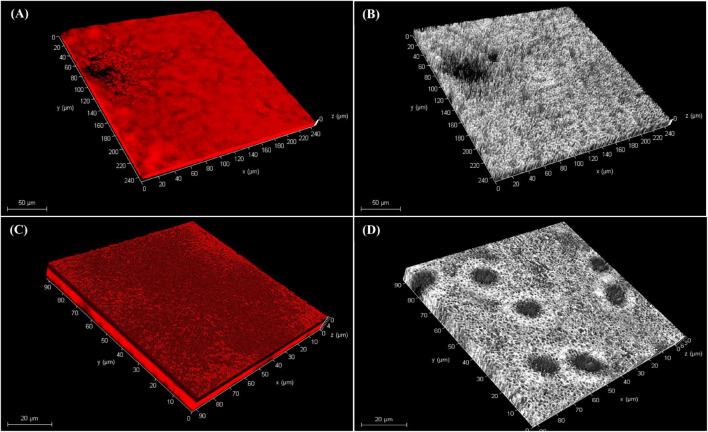
Confocal laser scanning microscopy of biofilm. Three-dimensional (3D) views of **(A,B)** M45 and **(C,D)** K96 biofilms grown for 48 h. Bacteria were stained with acridine orange and observed by CLSM.

CLSM microscopy observations revealed the thickness of the biofilms in the z-plane as 9 μm for K96 and 10 μm M45, respectively. The biofilm growth revealed that the cells of K96 and M45 attached to the glass surface and colonized the whole surface (Figure 5 and Supplementary Figure 4).

### Cell Surface Hydrophobicity Assay

The bacterial CSH plays a crucial role in bacterial aggregation which sequentially promotes bacterial dispersion and survival in soil and aid in microbial adhesion to plant surfaces during biofilm formation. In this study, 38.29% CSH was shown by K96 and 27.77% CSH by M45.

### Motility Assay

**FIGURE 6:**
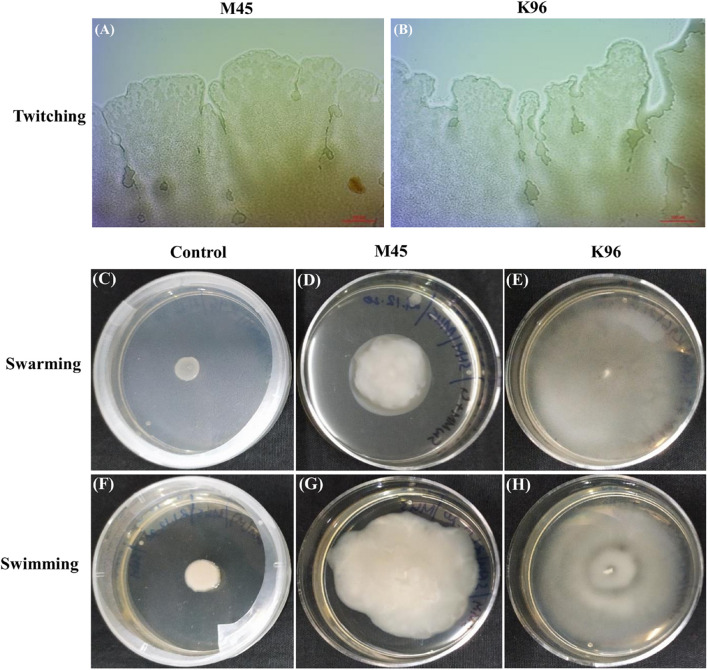
Motility assays. **(A,B)** Light microscopy of colony edges of M45 and K96 in twitching motility. Both M45 and K96 isolates produced a flat, widely spread, irregularly shaped colony at 100X magnification. **(C–E)** Swarming motility [NA plate with 5 g glucose/L and 0.5% (w/v) agar] and **(F–H)** Swimming motility [LB with 0.3% (w/v)] of Control, M45 and K96 incubated at 28°C for 48 h.

The bacterial motility was studied over time for bacterial isolates M45 and K96. Both the isolates were seen to exhibit twitching, swimming, and swarming motility. Isolates M45 and K96 isolates produced a flat, widely spread, irregularly shaped colony at 100X magnification in media-specific for twitching motility (Figures 6A,B). The isolates were seen to exhibit swarming motility with diameter 53 mm ± 1.2 and 45 mm ± 0.25 for K96 and M45, respectively, within a time period of 48 h (Figures 6C–E). Swimming assays revealed that M45 and K96 isolates are motile presenting concentric halos on 0.3% agar plates at 48 h (Figures 6F–H).

### Plant Growth Promotion Assay *in vivo* Using Selected Isolates

**FIGURE 7:**
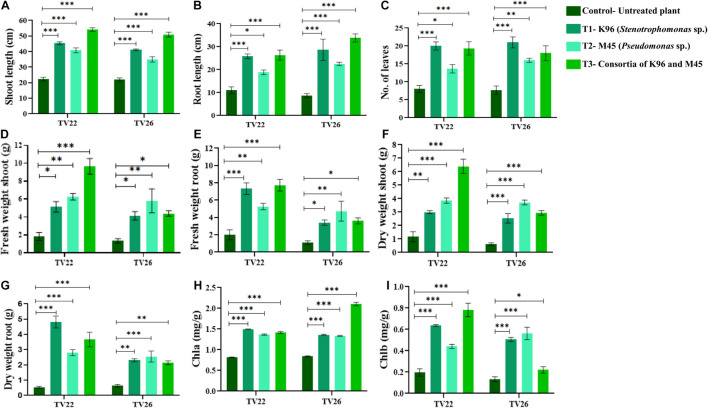
Evaluation of PGP experiment to show the effect of treatments of endophytic bacterial inoculum in two tea clones TV22 and TV26 for **(A)** shoot length; **(B)** root length; **(C)** number of leaves **(D)** fresh weight shoot; **(E)** fresh weight root; **(F)** dry weight shoot; **(G)** dry weight root; **(H)** chlorophyll a; and **(I)** chlorophyll b. Error bar represents the standard error of means of replicates (^∗^*p* < 0.05, ^∗∗^*p* < 0.01, ^∗∗∗^*p* < 0.001).

**FIGURE 8:**
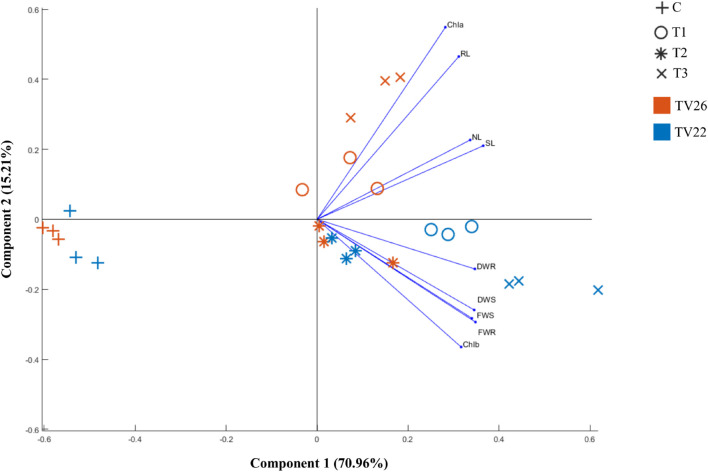
Principle component analysis biplot based on correlation matrices of the PGP dataset of growth parameters and treatment groups of tea clones TV22 and TV26. C- Control, Untreated plant; T1-Treatment 1, K96 (*Stenotrophomonas* sp.); T2- Treatment 2, M45 (*Pseudomonas* sp.); T3- Treatment 3, Consortia of K96 and M45.

Based on bonitur score, isolates with the top two scores namely K96 and M45 were selected to observe their effective contribution to promote plant growth in the treated plant group compared to the control group *in vivo* experiments in nursery conditions (Supplementary Figure 2). All the treated plant groups showed a significant (*p* < 0.05) increase in different plant growth-promoting vegetative parameters in comparison to the control plant group (Figure 7) on inoculation with the potent EnB isolates. PCA resulted in two major principal components (PC) with a variance of 70.96% (PC1) and 15.21% (PC2) on reduction of the dimension of the various PGP parameters (Figure 8). The analysis revealed a major distinction between treatment and control groups throughout the PC1. Among the treatment groups viz. T1 and T3 the interrelationship among the vegetative parameters in clone TV26 and TV22 were reflected by PC2 and PC1, respectively, where a minor distinction was observed in the case of the T1 group for both the clones. Likewise, PC1 represented a higher degree of correlation of parameters dry weight root and shoot (DWR and DWS), fresh weight shoot and root (FWS and FWR), and chlorophyll b (Chlb) toward clone TV22 in T1 and T2 group. Likewise, parameters chlorophyll a (Chla), root length (RL), number of leaves (NL), and shoot length (SL) were correlated mostly toward clone TV26 in both the treatment groups.

The level of growth in vegetative parameters of treated plants compared to untreated plants was measured by fold change analysis. T3 showed considerable increase in the number of fold change compared to T1 and T2 in TV22 tea clone with an increase in shoot length by 4.99-fold, root length by 3.93-fold, fresh shoot weight by 2.96-fold, dry shoot weight by 2.38-fold, fresh root weight by 2.51 fold, and dry root weight by 1.64-fold. However, T1 showed a maximum increase in the number of leaves of TV22 clone by 3.59-fold. In the case of clone TV26, the degree of growth promotion was more significant when treated with T2 that showed a 2.16 fold increase for fresh shoot weight, 1.63-fold for dry weight shoot, 1.86-fold for fresh root weight, and 0.94 fold for dry root weight. On the other hand, T3 showed maximum shoot length and root length with an increase of 4.85 and 4.65, respectively, and T1 showed a maximum increase in the number of leaves by 3.73 fold (Supplementary Figures 3A–F).

## Discussion

Over the past few decades to prevent food shortage worldwide and escalate crop yield, extensive amounts of chemical fertilizers, pesticides, and herbicides have been used that resulted in environmental hazards and is one of the major areas of concern in tea and other agricultural production (Sharma and Singhvi, 2017; Xie et al., 2019). It has become important to seek a substitute to take over these chemicals. The beneficial bacteria that inhabit inside plants can improve plant growth under natural and challenging conditions (Glick, 2012; Santoyo et al., 2016). They are capable of improving plant nutrient uptake, amending soil health, and priming plant defense to develop quality clones of tea with higher yield potential and confer sustainability in agriculture (Jain and Kumar, 2017). Consequently, understanding the EnB community associated with tea to delineate their function in PGP has become important. To endeavor beneficial effects on plant growth, PGPB has to colonize the endosphere after colonization in the rhizosphere. Many hypotheses including motility, attachment, plant-polymer degradation, production of growth phytohormones, phosphate solubilization, siderophore production, and evasion of plant defenses have been described to be involved in growth promotion by PGPB (Glick, 2014; Liu et al., 2017).

*Camellia sinensis* known for its therapeutic aid in several diseases is being widely consumed worldwide. Tea plants harbor a broad range of beneficial EnB which can be used as bioinoculants in promoting safe and sustainable agriculture. With the possibility to isolate diverse culturable EnB, leaf, and roots of five different tea clones were selected as variation in the endophytic community depends on the plant. Most t of the EnB isolates was recovered from root tissues as compared to leaf tissues which are unanimous with several studies that indicated the dominance of EnB in root tissues (Ma et al., 2013; Kandel et al., 2017).

PCR amplification followed by 16S rRNA gene sequencing and phylogenetic analysis determined the molecular identity and genetic diversity of these isolates. Similarity search using NCBI GenBank and EZ Taxon identified all the isolates at genus level showing 91–100% identity with reference sequences. Also, 16S rRNA gene sequencing is regarded as a robust technique for the identification of bacteria at genus and species level which differentiates between closely related bacterial species (Johnson et al., 2019). EnB were grouped into three phyla which consisted of 22 different genera that were grouped into six clusters based on phylogenetic analysis. Proteobacteria (*n* = 53, 50%) was most dominant which is represented by *Acinetobacter* sp., *Stenotrophomonas* sp., *Brevundimonas* sp., *Pseudomonas* sp., *Ochrobactrum* sp., *Alcaligenes* sp., *Serratia* sp., *Achromobacter* sp., and *Advenella* sp. followed by Firmicutes (*n* = 42, 39.62%) including *Lysinibacillus* sp., *Sporosarcina* sp., *Paenibacillus* sp., *Bacillus* sp., *Exiguobacterium* sp., *Planococcus* sp., and *Brevibacillus* sp. and Actinobacteria (*n* = 11, 10.37%) which is represented by *Rhodococcus* sp., *Janibacter* sp., *Curtobacterium* sp., *Streptomyces* sp., *Nocardia* sp., and *Microbacterium* sp. which is similar to the findings as reported by Verma et al. (2015). *Bacillus* was found to be the dominant genus (*n* = 31, 29.24%) followed by *Alcaligenes* (*n* = 18, 17.0%), *Pseudomonas* (*n* = 11,10.37%), and *Stenotrophomonas* (*n* = 10, 9.43%). *Bacillus* sp. was reported previously as dominant genera as an endophyte in different crops. In Pisarska and Pietr, (2015) and Passari et al. (2016) *Bacillus* was reported as the dominant genus among the EnB isolated from maize and *Clerodendrum colebrookianum* Walp which agrees with our findings.

For the judicious selection of potential indigenous PGP strains, the isolates were evaluated for a range of PGP traits by direct (include phosphate solubilization, production of IAA, ACC deaminase, and nitrogen fixation) and indirect (include siderophore, ammonia, and extracellular enzyme production) methods. All the isolates showed multifarious PGP traits which may be beneficial for the growth of the tea plant.

Phosphorus is an essential nutrient for plant growth which is required for numerous key plant functions inclusive of photosynthesis, nutrient uptake, and energy transfer, and so on. But it is unavailable to plants because most of the phosphorus exists in the soil in an insoluble form as organic phosphate or as mineral salts emerging as a major limiting factor in agricultural systems (Miller et al., 2010; Oteino et al., 2015). Thus, to determine the ability of EnB isolates to solubilize tri-calcium phosphate present in PKA and NBRIP medium quantitative and qualitative analysis was performed. A significant decrease in pH of culture medium due to production of organic acids confirmed phosphate solubilization by 30 of the isolates. As reported (Oteino et al., 2015) the acidity and concentration of soluble phosphate in the medium are inversely proportional. The phosphate solubilization values in this study fall in the range 2.79 ± 0.67- 81.42 ± 5.73 mg L^–1^ which is in agreement with the typical findings in PSB range from 10 to 800 mg L^–1^ as reported (Rodríguez and Fraga, 1999; Passari et al., 2016).

Auxin IAA production by beneficial bacteria is very essential for plant growth-promoting microorganisms as it is one of the most important phytohormones regulating growth and several developmental processes in plants (Egamberdieva et al., 2017). In the present study, noticeably more IAA was produced by isolates in presence of precursor L-tryptophan than previously reported by Hassan (2017) ranging from 4.1 to 23.4 μg mL^–1^ in *Teucrium polium* L. Similarly, Woźniak et al. (2019) reported IAA production by bacteria isolated from wheat, maize, rye, broad bean, burdock, and horsetail in the range 0.13–22.51 μg mL^–1^. Since IAA production is governed by multiple pathways, the amount of IAA produced may vary among isolates (Moliszewska and Nabrdalik, 2020). Studies revealed that most of the IAA producing EnB belong to phyla Proteobacteria, Firmicutes, and Actinobacteria (Nabrdalik et al., 2018; Hazarika and Thakur, 2020) which is in agreement with this study.

Production of ammonia by EnB potentially serves as a source for fixation of atmospheric nitrogen which stimulates plant growth and contributes to plant defense. Almost all the isolates produced ammonia among which *Bacillus* sp. produced a higher amount of ammonia which is similar with the report (Brígido et al., 2019; Kang et al., 2020) that can escalate plant biomass by the accumulation of nitrogen.

Siderophore production by plant-associated bacteria assists in plant growth by scavenging iron from the environment because of the low iron bioavailability (Ahmed and Holmström, 2014). The EnB isolates under iron limiting conditions produced a high amount of siderophore in this study. Siderophore production by *Brevundimonas* sp. has rarely been reported earlier which has been confirmed in our study, although it has been isolated from various sources such as rhizospheric soil of sugarcane, potato, and maize (Montañez et al., 2012; Kumar and Gera, 2014; Naqqash et al., 2020). Previous studies reported *Bacillus* sp. (Ferreira et al., 2019), *Pseudomonas* sp., *Stenotrophomonas* sp., *Acinetobacter* sp., and *Ochrobactrum* sp. (Yu et al., 2018; Ferreira et al., 2019) as producers of siderophore which is similar with our study.

Drought is one of the most common stresses induced in tea plants (Lin, 2011). Thus, one of the vital roles of beneficial bacteria associated with tea plants is to alleviate stress and promote growth. One such mechanism exploited by EnB is lowering plant ACC levels, a precursor of ethylene by the production of ACC deaminase (Sessitsch and Nowak, 2008). In the present study, 87.73% of isolates produced ACC deaminase which was much higher than reported by Borah et al. (2019). Production of other extracellular enzymes like cellulase, protease, and amylase plays a significant role in the decay of organic matter and plant growth promotion. In our study, *Bacillus* sp. exhibited the highest number of exoenzyme activities among tested enzymes, which is in accordance with the results reported (El-Deeb et al., 2012). Among other isolates *Pseudomonas* sp., *Stenotrophomonas* sp., *Ochrobactrum* sp., and *Alcaligenes* sp. produced all the enzymes tested. Among isolates, *Serratia* sp. and *Acinetobacter* sp. had cellulase and protease activity; *Streptomyces* sp. and *Brevundimonas* sp. displayed cellulase and amylase activity. Bacterial strains *Brevibacillus* sp., *Lysinibacillus* sp., and *Microbacterium* sp. showed only cellulase activity; *Achromobacter* sp. showed only amylase activity; and *Planococcus* sp., *Janibacter* sp., and *Paenibacillus* sp. produced only protease. This result is in accordance with the study of El-Deeb et al. (2012); Liu et al. (2016), and Passari et al. (2016). Many plant beneficial bacteria produces extracellular enzymes through indirect mechanism for PGP (Gupta et al., 2015). The production of hydrolytic enzymes by EnB emerges as important for colonization of plant roots and movement of bacteria into the interior of plants rendering help in plant growth (Al-Mallah et al., 1987; Quadt-Hallmann et al., 1997; Choubane et al., 2016).

As described by Chen et al. (2013) the ability of bacteria to form a stable biofilm for attachment and persistence on the plant root has been proven to be highly advantageous for plant growth promotion. Colonization of bacteria is crucial for plant-PGPB interactions to aid in plant growth. Thus, for biofilm formation and competitive colonization in the plant-root system bacterial motility plays an important role (Harshey, 2003; Danhorn and Fuqua, 2007). Motility and biofilm formation exhibited by the tea-associated bacteria M45 and K96 denotes their potentiality as PGPB and may colonize and establish on plant root system with ease, aiding in plant growth promotion.

Several reports have shown endophytic bacteria can boost plant biomass in different plants rendering multifarious advantages to host plants (Dutta and Thakur, 2017; Finkel et al., 2017). The PGP experiment was performed in an established natural condition using non-sterile soil. The soil microflora associated with tea is diverse and complex which portrays a substantial challenge in tea cultivation. To assess the efficacy of the indigenous bacterial inoculum and to constitute a compatible interaction with the host the treated tea plants were compared with untreated tea plants in the same condition. Multifarious experiments on PGP traits and bonitur scale assessment ameliorated the selection of potent isolates which augmented all growth parameters tested in the nursery experiment. The most promising effect on the tea plants tested were displayed by consortia of the isolates than the individual inoculum. The multivariate PCA analysis showed accord with the treatment and vegetative growth parameters. The efficiency of the selected isolates was further confirmed as the treated plants deviated significantly from the untreated plants. This was validated by two-way ANOVA analysis. Therefore, the bacteria inoculums utilized in this nursery experiment were assumed to show synergistic PGP effect, colonization, and competence with the other native microbial population in the treated tea clones.

*In vivo*, the growth of the tea plants is not directly related to the growth-promoting traits such as IAA production, phosphate solubilization, ACC deaminase production, and so on. The specificity of the bacteria’s interaction with the plants influenced the growth-promotion. The efficiency of the PGP strains was possibly dependent on the genotype of the host plant or the bacterial genotype. Thus, the genetic factors of both partners appear to influence plant colonization and growth promotion by the EnB (Afzal et al., 2019).

## Conclusion

Endophytic bacteria residing within the host plant are potential natural bioresources aiding in plant growth promotion. The exploration of endophytes indigenous to tea plants is limited as compared to endophytes from various agricultural systems. This current study helped in establishing the benefits of endophytes associated with tea clones endowed with plant growth-promoting traits, motility, and biofilm-forming ability and suggesting its applicability in tea gardens and other agricultural crops as biofertilizer or as an alternative for chemical fertilizers and pesticides, thereby promoting sustainability. However, further research is needed to establish the colonization of the endophytes within the host and study the correlation with the native soil microflora.

## Data Availability Statement

The datasets presented in this study can be found in online repositories. The names of the repository/repositories and accession number(s) can be found in the article/Supplementary Material.

## Author Contributions

SH conceptualized, planned, designed, and conducted the laboratory and field experiments, acquired and analyzed the data, interpreted the results, and wrote and reviewed the original draft. KS contributed to data analysis. AB helped in an experimental setup. DT conceptualized and supervised the research work and reviewed and edited the original draft. All authors have read and approved the final manuscript.

## Conflict of Interest

The authors declare that the research was conducted in the absence of any commercial or financial relationships that could be construed as a potential conflict of interest.

## Publisher’s Note

All claims expressed in this article are solely those of the authors and do not necessarily represent those of their affiliated organizations, or those of the publisher, the editors and the reviewers. Any product that may be evaluated in this article, or claim that may be made by its manufacturer, is not guaranteed or endorsed by the publisher.
