# Supplementary material for: Prospecting Endophytic Bacteria Endowed With Plant Growth Promoting Potential Isolated From Camellia sinensis
Source: Front Microbiol. 2021 Sep 30;12:738058. doi: 10.3389/fmicb.2021.738058 (PMC8515050; doi:10.3389/fmicb.2021.738058)
Supplement: Supplementary file 1 [file Data_Sheet_1.docx]

**Supplementary materials**

**Supplementary Figure 1**. Log colony forming unit per gram of endophytic bacteria isolated from leaf and roots of five different tea clones.

**
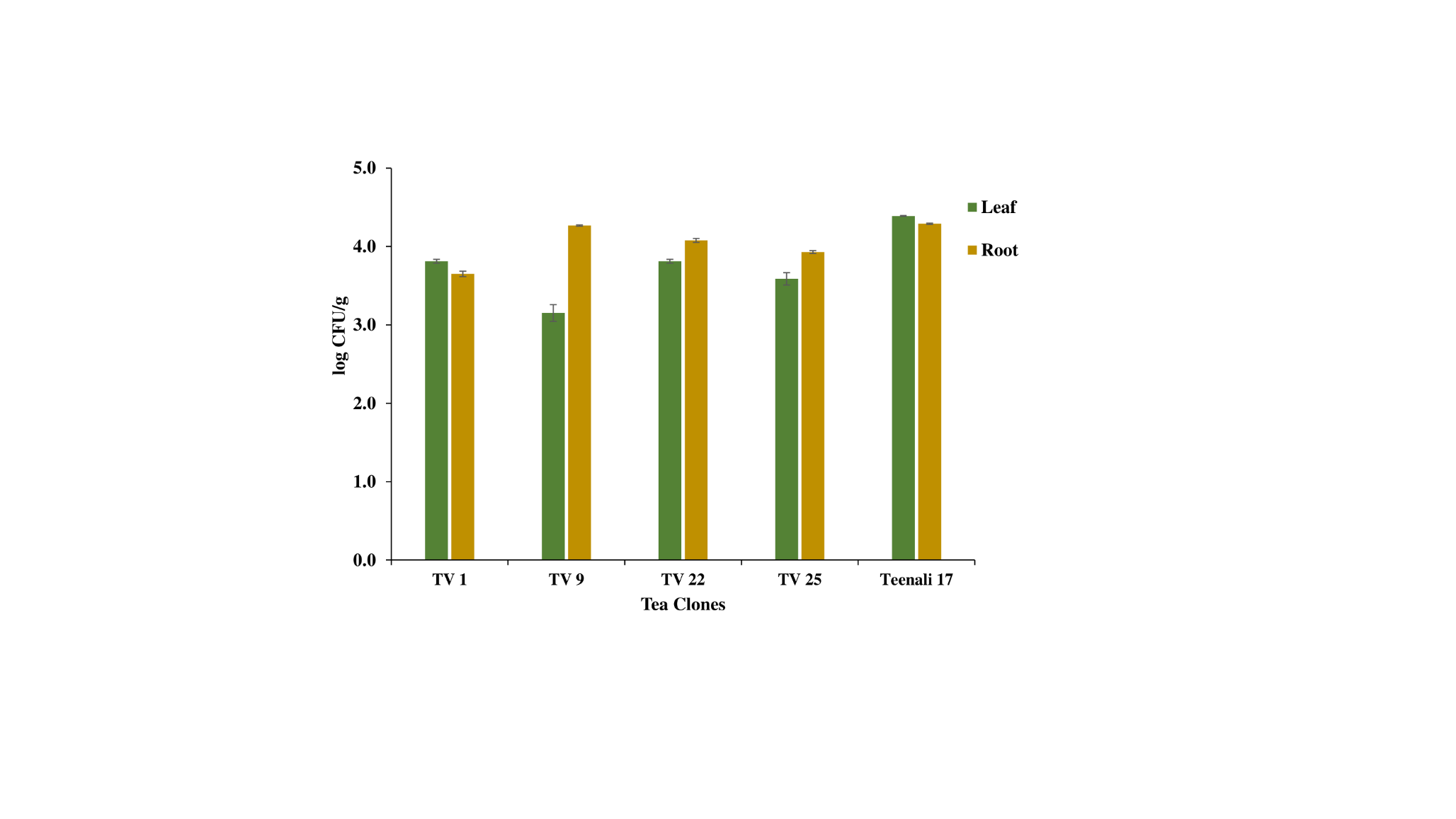
**

**Supplementary Figure 2**. *In vivo* plant growth promotion experiment in nursey condition on commercially cultivated tea clones **(A)**TV26 and **(B)** TV22. **(C)** Comparison of treated tea plant with untreated tea plant on addition of bacterial inoculum **(D)** Comparison of root system of treated tea plant with untreated tea plant on addition of bacterial inoculum.

Control- Untreated plant; Treatment 1 (T1)- K96 (*Stenotrophomonas* sp.); Treatment 2 (T2)- M45 (*Pseudomonas* sp.); Treatment 3 (T3)- Consortia of K96 and M45.


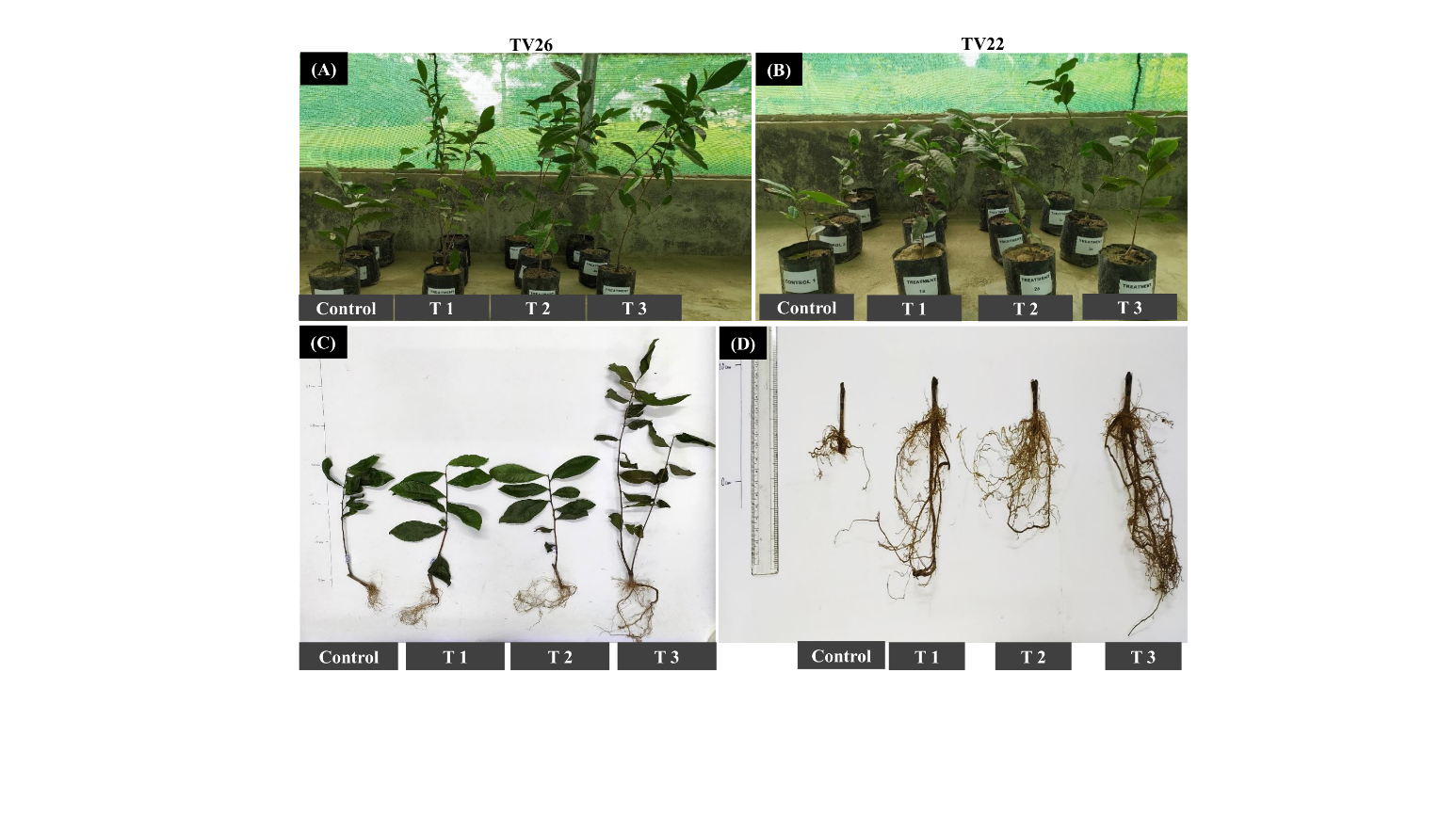


**Supplementary Figure 3.** Fold change analysis of tea clones TV22, and TV26 treated with endophytic bacterial strains

**Tea clone TV22**

1. **
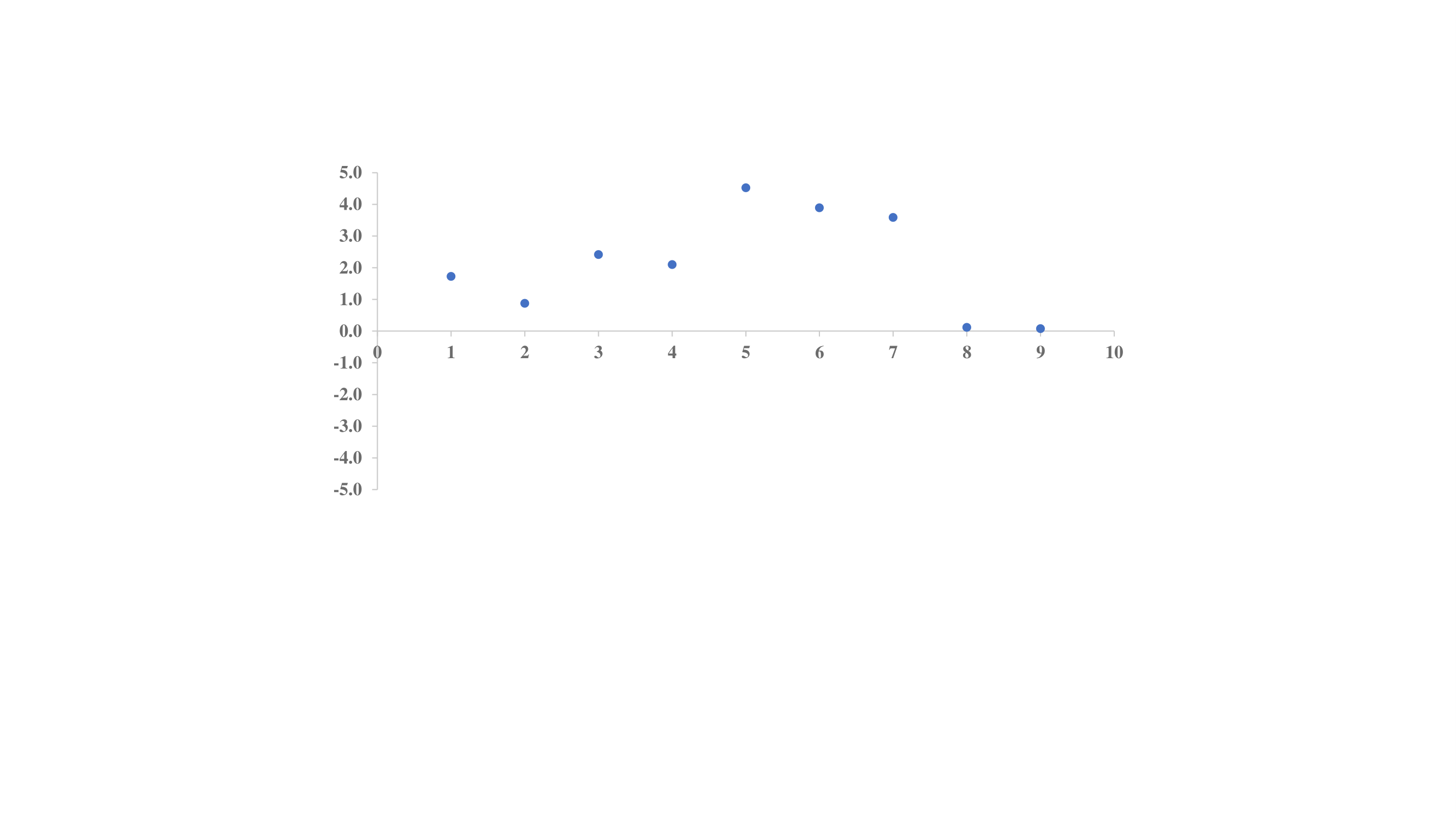
Treatment T1 (K96)**

| **Growth parameters** | **Fold change (FC)** | **Log2(FC)** |
| --- | --- | --- |
| Fresh weight shoot | 3.300 | 1.722 |
| Dry weight shoot | 1.833 | 0.874 |
| Fresh weight root | 5.333 | 2.415 |
| Dry weight root | 4.283 | 2.099 |
| Shoot Length | 23.000 | 4.524 |
| Root Length | 14.833 | 3.891 |
| Number of leaves | 12.000 | 3.585 |
| Chlorophyll a | 1.083 | 0.115 |
| Chlorophyll b | 1.053 | 0.074 |

1. **
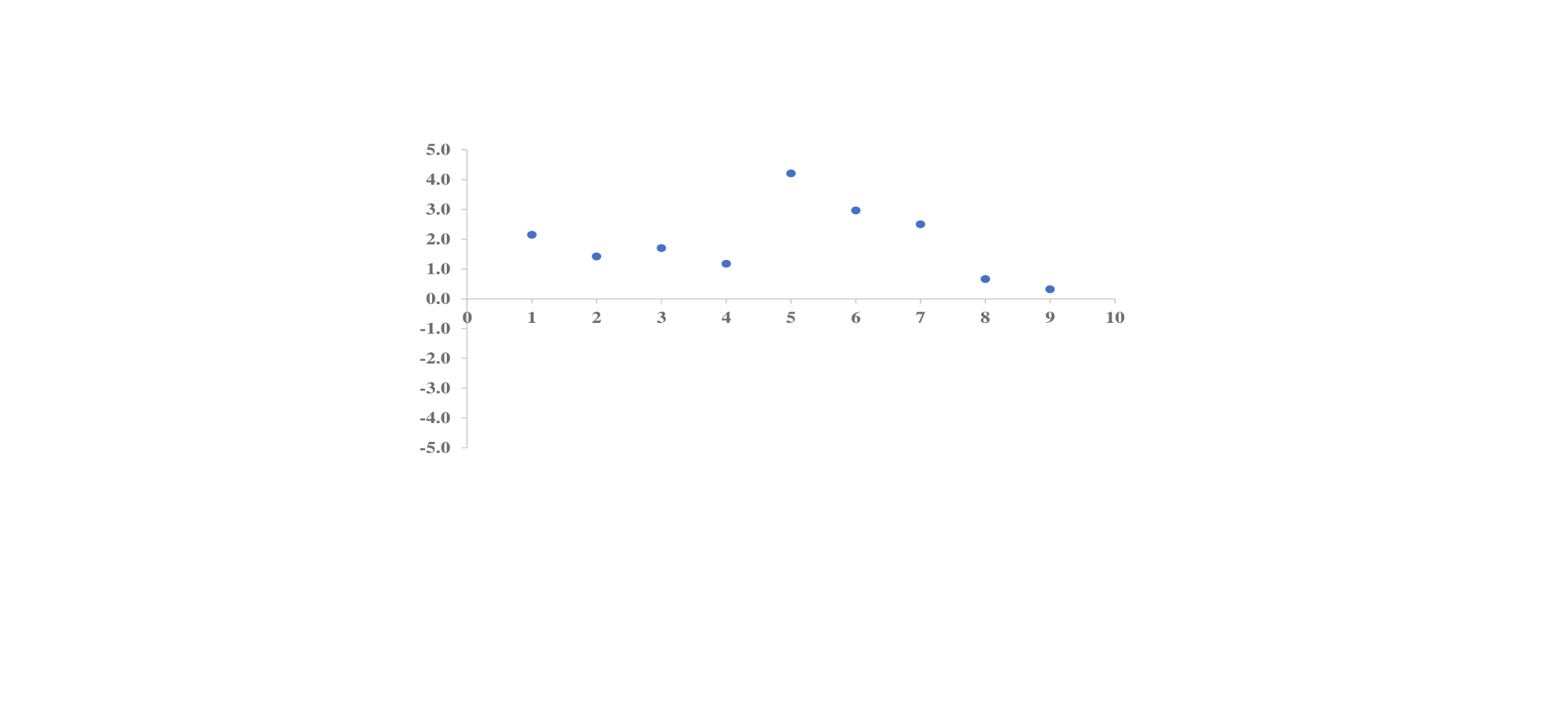
Treatment T2 (M45)**

| **Growth parameters** | **Fold change**  **(FC)** | **Log2(FC)** |
| --- | --- | --- |
| Fresh weight shoot | 4.430 | 2.147 |
| Dry weight shoot | 2.687 | 1.426 |
| Fresh weight root | 3.267 | 1.708 |
| Dry weight root | 2.267 | 1.181 |
| Shoot Length | 18.500 | 4.209 |
| Root Length | 7.833 | 2.970 |
| Number of leaves | 5.667 | 2.503 |
| Chlorophyll a | 1.583 | 0.662 |
| Chlorophyll b | 1.253 | 0.325 |

1. **
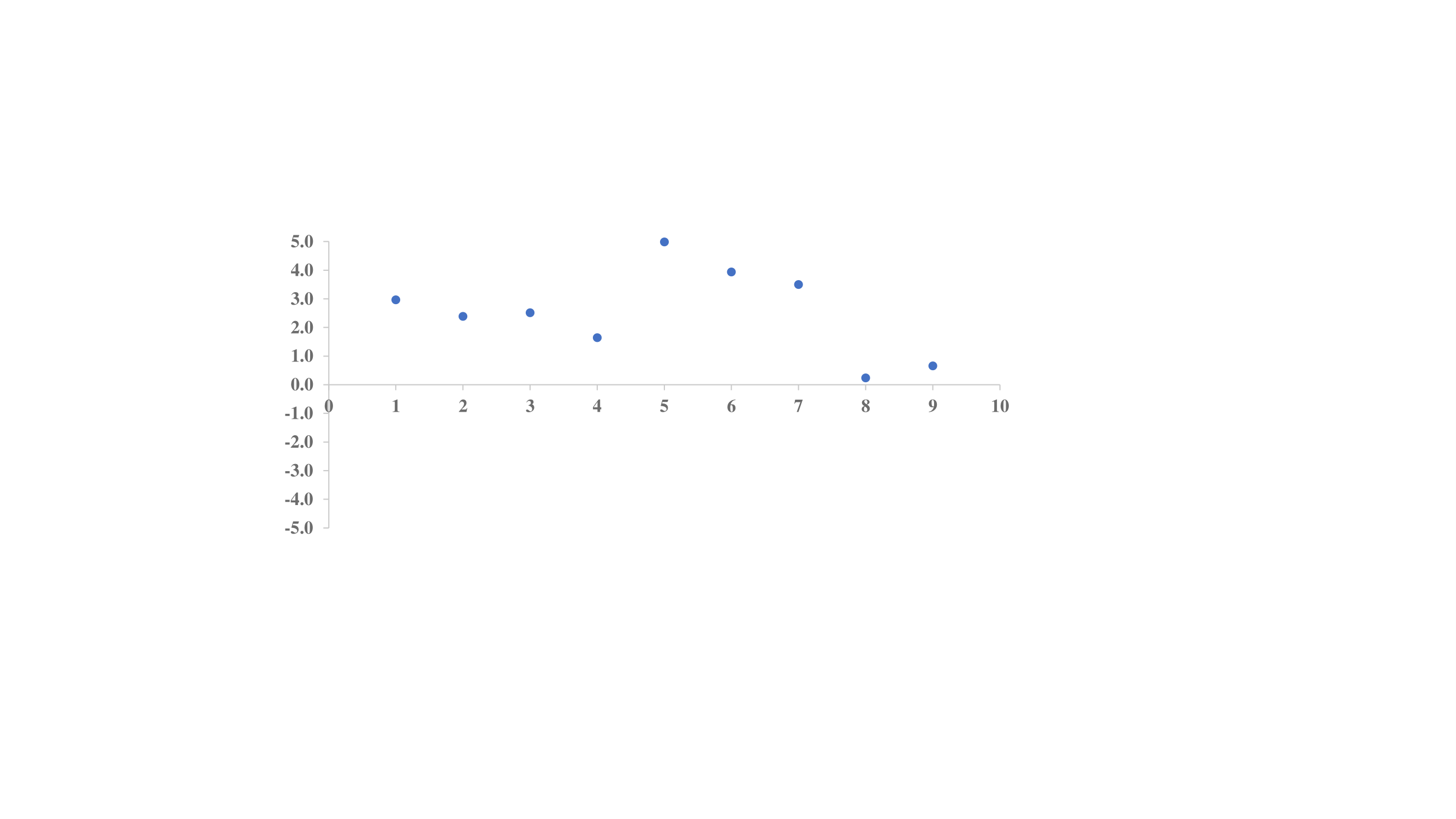
 Treatment T3 (K96+M45)**

| **Growth parameters** | **Fold change**  **(FC)** | **Log2(FC)** |
| --- | --- | --- |
| Fresh weight shoot | 7.830 | 2.969 |
| Dry weight shoot | 5.233 | 2.388 |
| Fresh weight root | 5.733 | 2.519 |
| Dry weight root | 3.133 | 1.648 |
| Shoot Length | 31.833 | 4.992 |
| Root Length | 15.333 | 3.939 |
| Number of leaves | 11.333 | 3.503 |
| Chlorophyll a | 1.183 | 0.242 |
| Chlorophyll b | 1.585 | 0.664 |

**Tea clone TV26**

1. **
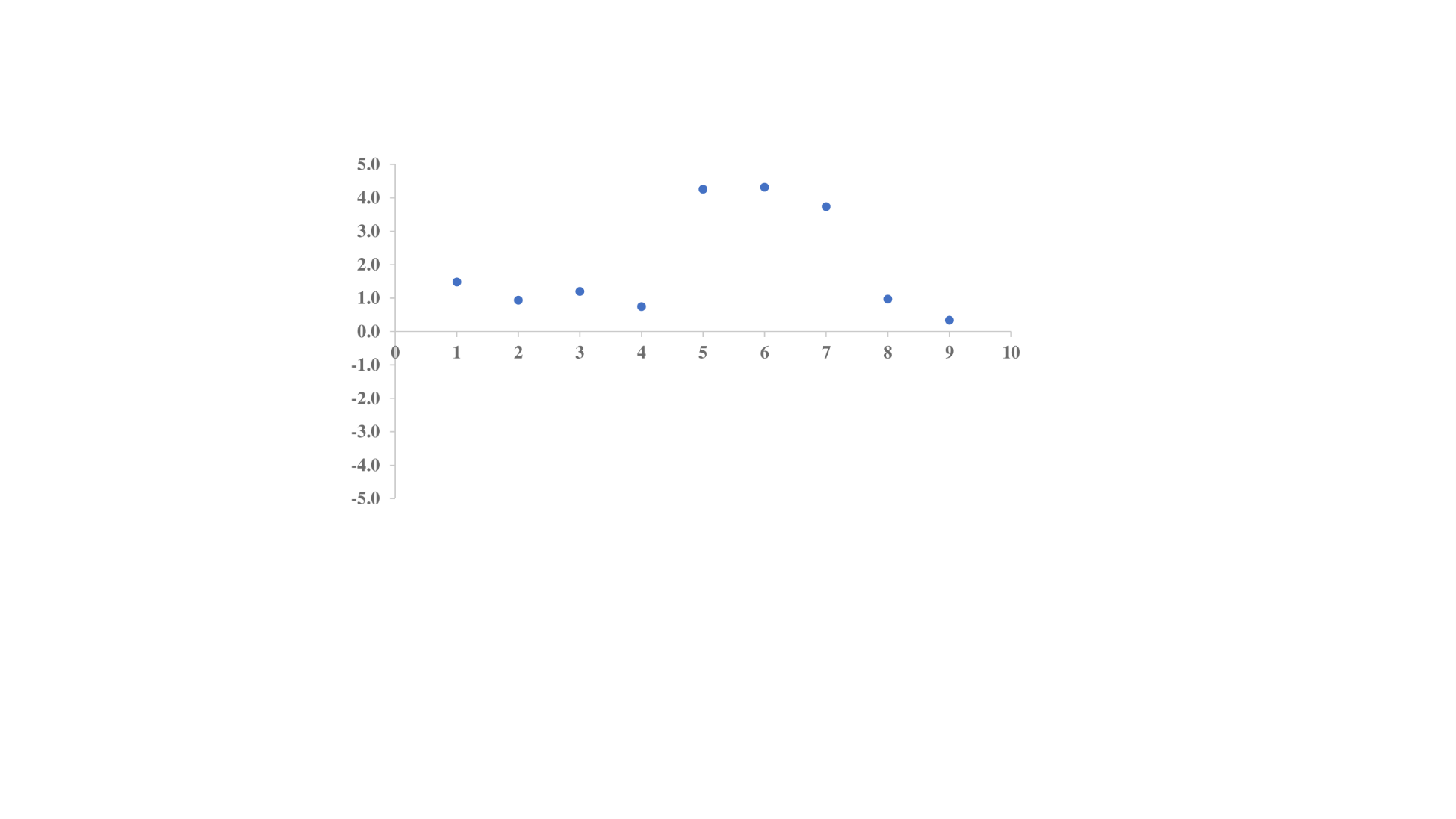
Treatment T1 (K96)**

| **Growth parameters** | **Fold change**  **(FC)** | **Log2(FC)** |
| --- | --- | --- |
| Fresh weight shoot | 2.800 | 1.485 |
| Dry weight shoot | 1.917 | 0.939 |
| Fresh weight root | 2.300 | 1.202 |
| Dry weight root | 1.677 | 0.746 |
| Shoot Length | 19.167 | 4.261 |
| Root Length | 20.000 | 4.322 |
| Number of leaves | 13.333 | 3.737 |
| Chlorophyll a | 1.960 | 0.971 |
| Chlorophyll b | 1.266 | 0.341 |

1. **
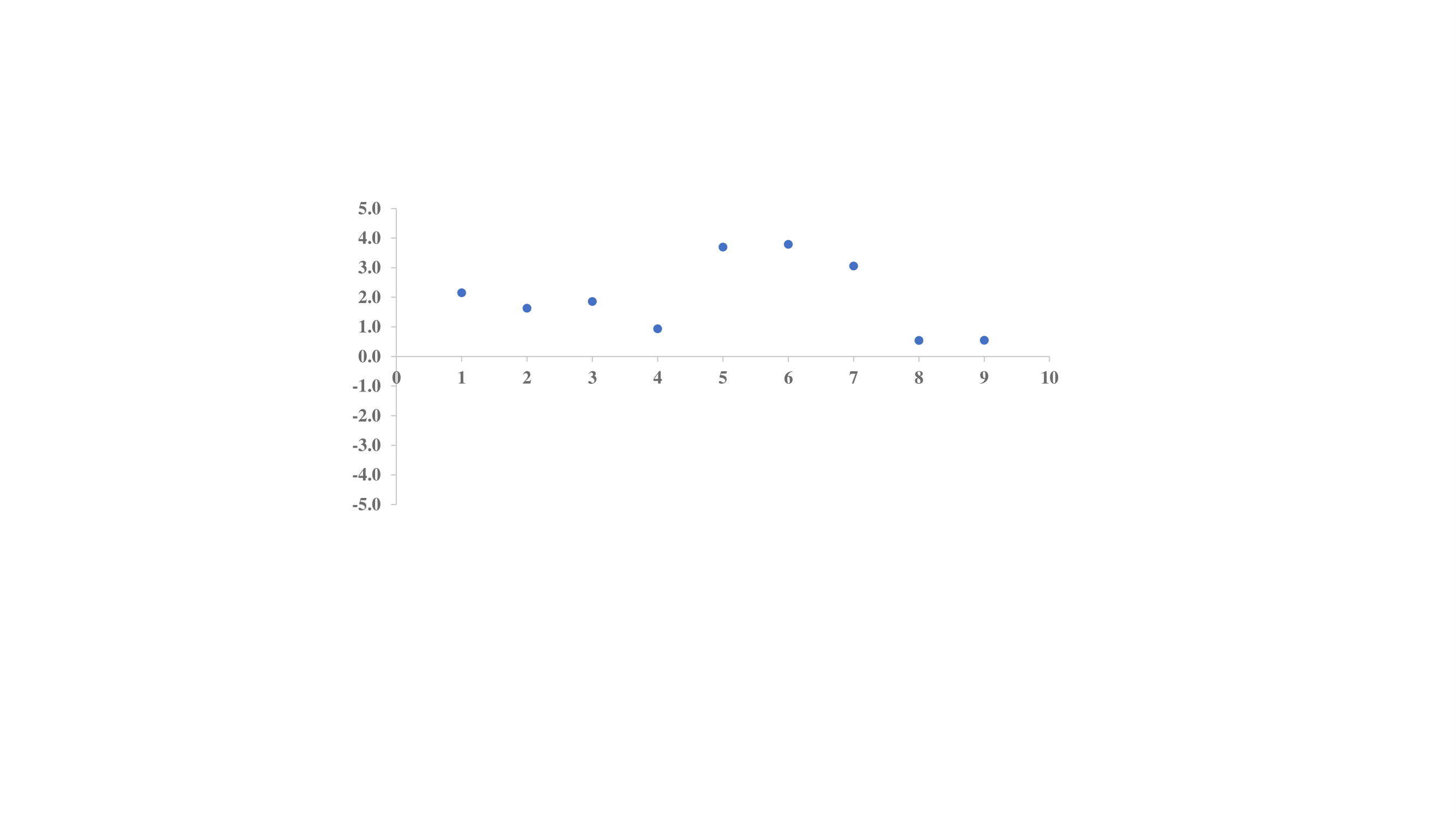
Treatment T2 (M45)**

| **Growth parameters** | **Fold change**  **(FC)** | **Log2(FC)** |
| --- | --- | --- |
| Fresh weight shoot | 4.467 | 2.159 |
| Dry weight shoot | 3.100 | 1.632 |
| Fresh weight root | 3.633 | 1.861 |
| Dry weight root | 1.913 | 0.936 |
| Shoot Length | 13.000 | 3.700 |
| Root Length | 13.833 | 3.790 |
| Number of leaves | 8.333 | 3.059 |
| Chlorophyll a | 1.460 | 0.546 |
| Chlorophyll b | 1.466 | 0.552 |

1. **
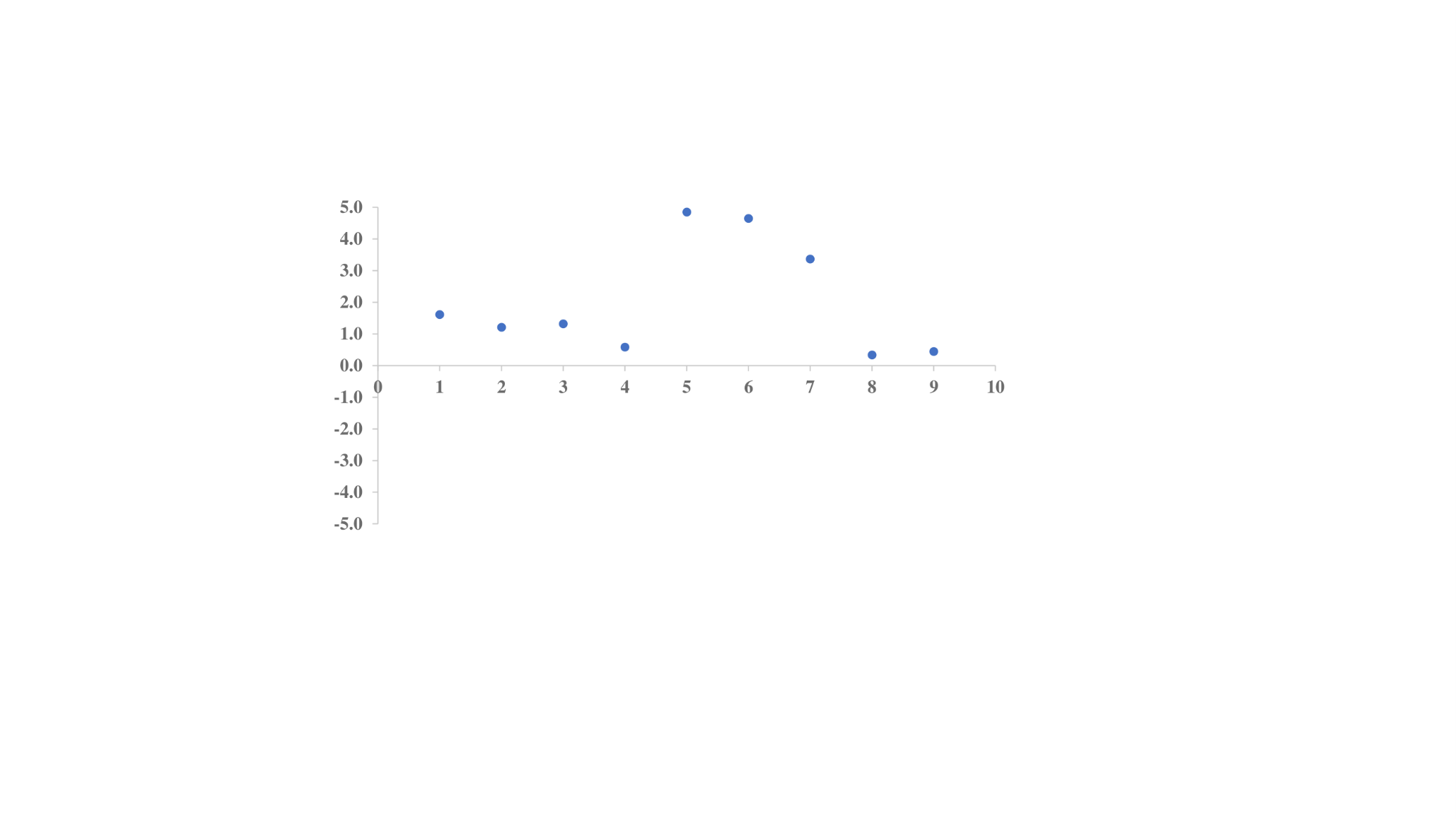
Treatment T3 (K96+M45)**

| **Growth parameters** | **Fold change**  **(FC)** | **Log2(FC)** |
| --- | --- | --- |
| Fresh weight shoot | 3.067 | 1.617 |
| Dry weight shoot | 2.317 | 1.212 |
| Fresh weight root | 2.500 | 1.322 |
| Dry weight root | 1.500 | 0.585 |
| Shoot Length | 28.900 | 4.853 |
| Root Length | 25.167 | 4.653 |
| Number of leaves | 10.333 | 3.369 |
| Chlorophyll a | 1.262 | 0.336 |
| Chlorophyll b | 1.366 | 0.450 |

**Supplementary Figure 4.** CLSM images of biofilm formation by **(A)** M45 and **(B)** K96 at 48 h with depth coding and scale bar.

**
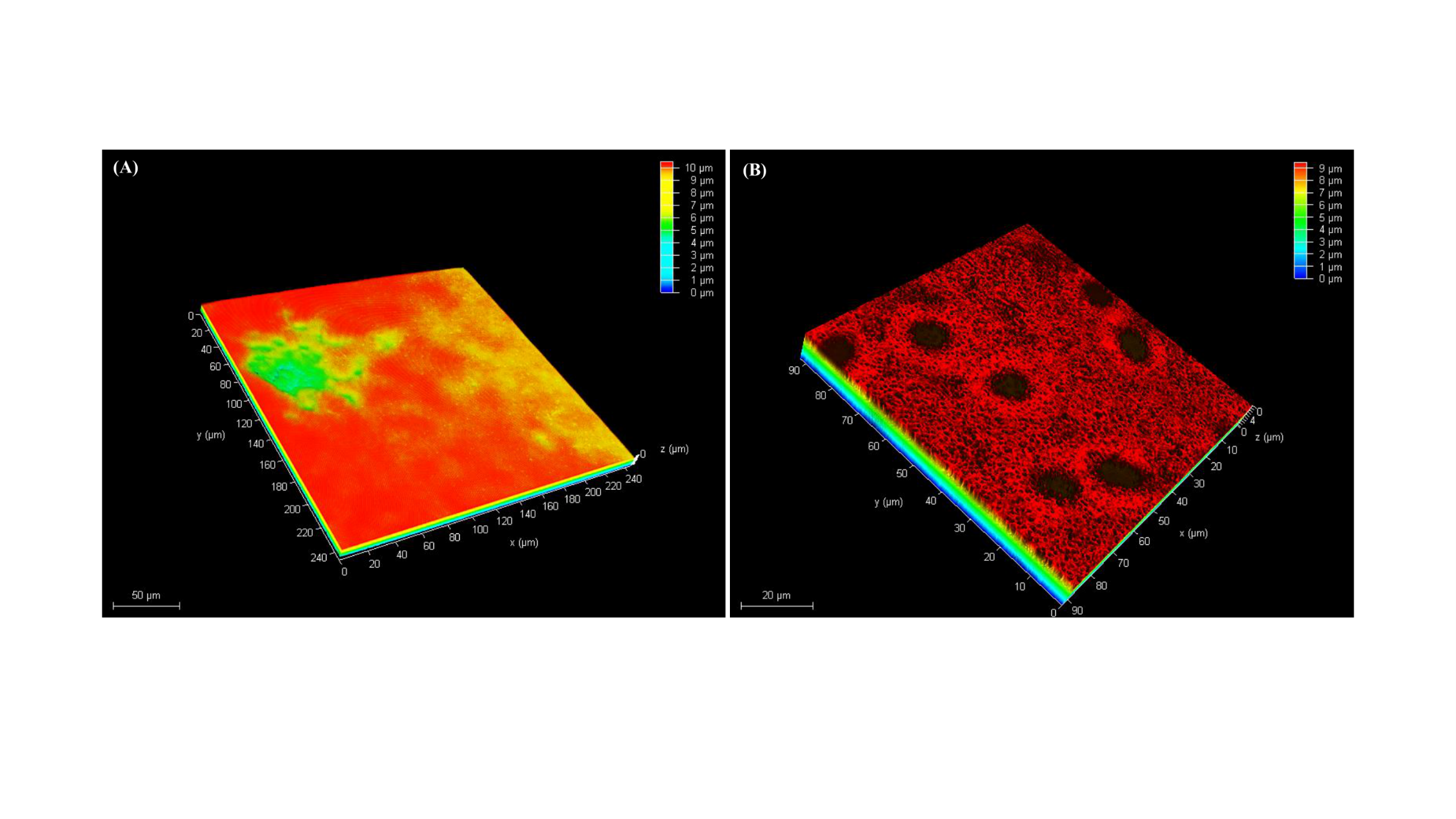
**

**Supplementary Table 1.** Identification of bacterial endophytes isolated from Tea clones based on 16S rRNA gene sequences similarity

| Isolate No. | Tea clone | Tissue | Media | Organism Name | Type strain | % Of similarity | Accession No. | Phylum |
| --- | --- | --- | --- | --- | --- | --- | --- | --- |
| K1 | Teenali 17 | Leaf | ISP7 | *Acinetobacter* sp. | *Acinetobacter lwoffii* strain JCM 6840 | 99.71 | MZ008003 | Proteobacteria |
| K2 | Teenali 17 | Leaf | AIA | *Stenotrophomonas* sp. | *Stenotrophomonas maltophilia strain* ATCC 16367 | 99.86 | MN493874.1 | Proteobacteria |
| K4 | Teenali 17 | Leaf | ISP7 | *Brevundimonas* sp. | *Brevundimonas naejangsanensis* strain BIO-TAS2-2 | 99.85 | MN493875.1 | Proteobacteria |
| K7 | Teenali 17 | Leaf | ISP3 | *Stenotrophomonas* sp. | *Stenotrophomonas pavanii* strain LMG 25348 | 99.51 | MN493876.1 | Proteobacteria |
| K8 | Teenali 17 | Leaf | NA | *Acinetobacter* sp. | *Acinetobacter radioresistens* strain NBRC 102413 | 99.37 | MZ008004 | Proteobacteria |
| K9 | Teenali 17 | Leaf | NA | *Ochrobactrum* sp. | *Ochrobactrum anthropi* strain NBRC 102587 | 99.42 | MN493877.1 | Proteobacteria |
| K10 | Teenali 17 | Leaf | ISP7 | *Ochrobactrum* sp. | *Ochrobactrum anthropi* strain NBRC 102587 | 99.71 | MN493878.1 | Proteobacteria |
| K11 | Teenali 17 | Leaf | AIA | *Lysinibacillus* sp. | *Lysinibacillus fusiformis* strain NBRC 15717 | 99.86 | MN493879.1 | Firmicutes |
| K12 | Teenali 17 | Leaf | SA | *Stenotrophomonas* sp. | *Stenotrophomonas rhizophilia* strain e-p10 | 98.61 | MN493880.1 | Proteobacteria |
| K13 | Teenali 17 | Leaf | SA | *Alcaligenes* sp. | *Alcaligenes faecalis* strain NBRC 13111 | 99.79 | MN493881.1 | Proteobacteria |
| K14 | Teenali 17 | Leaf | AIA | *Alcaligenes* sp. | *Alcaligenes faecalis* strain NBRC 13111 | 99.65 | MN493882.1 | Proteobacteria |
| K15 | Teenali 17 | Leaf | SA | *Sporosarcina* sp. | *Sporosarcina globispora* strain NBRC 16082 | 99 | MN493883.1 | Firmicutes |
| K16 | Teenali 17 | Leaf | ISP7 | *Paenibacillus* sp. | *Paenibacillus taichungensis* strain BCRC 17757 | 99.72 | MN493884.1 | Firmicutes |
| K17 | Teenali 17 | Leaf | SA | *Alcaligenes* sp. | *Alcaligenes faecalis* strain IAM 12369 | 99.58 | MW898446 | Proteobacteria |
| K18 | Teenali 17 | Leaf | AIA | *Bacillus* sp. | *Bacillus* *subtilis* strain DSM 10 | 100 | MN493885.1 | Firmicutes |
| K19 | Teenali 17 | Leaf | AIA | *Alcaligenes* sp. | *Alcaligenes faecalis* strain NBRC 13111 | 99.51 | MN493886.1 | Proteobacteria |
| K20 | Teenali 17 | Leaf | NA | *Alcaligenes* sp. | *Alcaligenes faecalis* strain NBRC 13111 | 99.44 | MN493887.1 | Proteobacteria |
| K21 | Teenali 17 | Root | PA | *Sporosarcina* sp. | *Sporosarcina koreensis* strain F73 | 99.45 | MN493888.1 | Firmicutes |
| K22 | Teenali 17 | Root | PA | *Sporosarcina* sp. | *Sporosarcina* *koreensis* strain F73 | 99.72 | MN493889.1 | Firmicutes |
| K25 | Teenali 17 | Root | PKA | *Bacillus* sp. | *Bacillus licheniformis* strain DSM 13 | 99.93 | MN493890.1 | Firmicutes |
| K26 | Teenali 17 | Root | PKA | *Bacillus* sp. | *Bacillus wiedmannii* strain FSL W8-0169 | 99.86 | MN577364.1 | Firmicutes |
| K27 | TV9 | Root | PA | *Rhodococcus* sp. | *Rhodococcus qingshengii* strain djl-6-2 | 99.29 | MN493891.1 | Actinobacteria |
| K30 | TV9 | Root | PA | *Bacillus* sp. | *Bacillus subtilis* strain DSM 10 | 99.93 | MW898447 | Firmicutes |
| K31 | TV9 | Root | SA | *Bacillus* sp. | *Bacillus subtilis* strain DSM 10 | 99.76 | MN493892.1 | Firmicutes |
| K33 | TV9 | Root | ISP7 | *Streptomyces* sp. | *Streptomyces sampsonii* strain ATCC 25495 | 93.16 | MW898680 | Actinobacteria |
| K34 | TV9 | Root | AIA | *Streptomyces* sp. | *Streptomyces sampsonii* strain ATCC 25495 | 98.8 | MW898681 | Actinobacteria |
| K36 | TV1 | Leaf | PKA | *Pseudomonas* sp. | *Pseudomonas putida* strain NBRC 14164 | 98.6 | MN493895.1 | Proteobacteria |
| K37 | TV1 | Root | PKA | *Pseudomonas* sp. | *Pseudomonas putida* strain ATCC 12633 | 99.72 | MN493896.1 | Proteobacteria |
| K38 | TV1 | Root | PKA | *Pseudomonas* sp. | *Pseudomonas putida* strain NBRC 14164 | 99.65 | MN493897.1 | Proteobacteria |
| K39 | TV1 | Root | AIA | *Ochrobactrum* sp. | *Ochrobactrum pseudogrignonense* strain CCUG 30717 | 99.63 | MN493898.1 | Proteobacteria |
| K41 | Teenali 17 | Leaf | PKA | *Pseudomonas* sp. | *Pseudomonas putida* strain NBRC 14164 | 99.44 | MN493899.1 | Proteobacteria |
| K42 | Teenali 17 | Leaf | SA | *Bacillus* sp. | *Bacillus subtilis* strain JCM 1465 | 99.86 | MN493900.1 | Firmicutes |
| K44 | Teenali 17 | Leaf | ISP7 | *Alcaligenes* sp. | *Alcaligenes faecalis* strain NBRC 13111 | 98.81 | MN493901.1 | Proteobacteria |
| K45 | Teenali 17 | Leaf | NA | *Alcaligenes* sp. | *Alcaligenes faecalis* strain NBRC 13111 | 99.51 | MN493902.1 | Proteobacteria |
| K46 | Teenali 17 | Leaf | PKA | *Alcaligenes* sp. | *Alcaligenes faecalis* strain NBRC 13111 | 99.44 | MN493903.1 | Proteobacteria |
| K49 | Teenali 17 | Root | PKA | *Exiguobacterium* sp. | *Exiguobacterium* *aquaticum* strain IMTB-3094 | 99.52 | MN493904.1 | Firmicutes |
| K50 | Teenali 17 | Root | PKA | *Bacillus* sp. | *Bacillus* *subtilis* strain DSM 10 | 99.93 | MN493905.1 | Firmicutes |
| K51 | Teenali 17 | Root | PKA | *Stenotrophomonas* sp. | *Stenotrophomonas* *pavanii* strain LMG 25348 | 98.47 | MN493906.1 | Proteobacteria |
| K53 | TV25 | Root | AIA | *Stenotrophomonas* sp. | *Stenotrophomonas* *maltophilia* strain IAM 12423 | 99.85 | MN493907.1 | Proteobacteria |
| K54 | TV25 | Root | PKA | *Brevundimonas* sp. | *Brevundimonas* *naejangsanensis* strain BIO-TAS2-2 | 95.07 | MN493908.1 | Proteobacteria |
| K55 | TV25 | Root | PKA | *Stenotrophomonas* sp. | *Stenotrophomonas* *maltophilia* strain ATCC 13637 | 99.79 | MW898448 | Proteobacteria |
| K56 | TV25 | Root | PA | *Stenotrophomonas* sp. | *Stenotrophomonas* *maltophilia* strain ATCC 13637 | 99.86 | MN493909.1 | Proteobacteria |
| K57 | TV25 | Root | NA | *Bacillus* sp. | *Bacillus* *wiedmannii* strain FSL W8-0169 | 100 | MN493910.1 | Firmicutes |
| K58 | TV25 | Leaf | PKA | *Brevundimonas* sp. | *Brevundimonas* *diminuta* strain LMG 2089 | 91.65 | MN493911.1 | Proteobacteria |
| K59 | TV25 | Leaf | AIA | *Brevundimonas* sp. | *Brevundimonas* *diminuta* strain JCM 2788 | 99.7 | MN493912.1 | Proteobacteria |
| K61 | TV9 | Root | NA | *Serratia* sp. | *Serratia* *marcescens* strain NBRC 102204 | 99.65 | MN493913.1 | Proteobacteria |
| K63 | TV9 | Root | NA | *Bacillus* sp. | *Bacillus* *subtilis* strain NBRC 101239 | 99.79 | MN493914.1 | Firmicutes |
| K64 | TV9 | Root | NA | *Bacillus* sp. | *Bacillus* *subtilis* strain NBRC 101239 | 99.86 | MN493915.1 | Firmicutes |
| K66 | TV9 | Root | NA | *Sporosarcina* sp. | *Sporosarcina* *globispora* strain NBRC 16082 | 96.96 | MN493916.1 | Firmicutes |
| K67 | TV9 | Root | ISP7 | *Achromobacter* sp. | *Achromobacter* *marplatensis* strain LMG 3458 | 99.65 | MN493917.1 | Proteobacteria |
| K68 | TV9 | Root | PA | *Achromobacter* sp. | *Achromobacter* *marplatensis* strain LMG 5911 | 99.79 | MN493918.1 | Proteobacteria |
| K69 | Teenali 17 | Root | AIA | *Alcaligenes* sp. | *Alcaligenes faecalis* strain NBRC 13111 | 99.44 | MN493919.1 | Proteobacteria |
| K70 | Teenali 17 | Root | PA | *Alcaligenes* sp. | *Alcaligenes faecalis* strain NBRC 13111 | 99.51 | MN493920.1 | Proteobacteria |
| K71 | Teenali 17 | Root | NA | *Paenibacillus* sp. | *Paenibacillus* *taichungensis* strain BCRC 17757 | 99.72 | MN493921.1 | Firmicutes |
| K72 | Teenali 17 | Root | NA | *Bacillus* sp. | *Bacillus* *pseudomycoides* strain NBRC 101232 | 98.88 | MN577365.1 | Firmicutes |
| K73 | Teenali 17 | Root | NA | *Bacillus* sp. | *Bacillus cereus* strain ATCC 14579 | 99.86 | MN493922.1 | Firmicutes |
| K74 | Teenali 17 | Root | NA | *Bacillus* sp. | *Bacillus pseudomycoides* strain NBRC 101232 | 98.88 | MN493923.1 | Firmicutes |
| K75 | Teenali 17 | Root | PKA | *Bacillus* sp. | *Bacillus cereus* strain IAM 12605 | 99.93 | MN493924.1 | Firmicutes |
| K76 | Teenali 17 | Root | SA | *Alcaligenes* sp. | *Alcaligenes faecalis* strain NBRC 13111 | 99.51 | MN493925.1 | Proteobacteria |
| K78 | Teenali 17 | Root | PKA | *Nocardia* sp. | *Nocardia globerula* strain DSM 44596 | 99.41 | MW898682 | Actinobacteria |
| K80 | Teenali 17 | Root | PKA | *Bacillus* sp. | *Bacillus kochii* strain WCC 4582 | 99.44 | MW905606 | Firmicutes |
| K81 | Teenali 17 | Root | PKA | *Bacillus* sp. | *Bacillus kochii* strain WCC 4582 | 99.51 | MN577367.1 | Firmicutes |
| K82 | TV22 | Leaf | NA | *Bacillus* sp. | *Bacillus subtilis* strain NBRC 13719 | 99.93 | MN577368.1 | Firmicutes |
| K84 | TV22 | Leaf | PA | *Bacillus* sp. | *Bacillus aryabhattai* strain B8W22 | 99.86 | MN577369.1 | Firmicutes |
| K85 | TV22 | Leaf | NA | *Planococcus* sp. | *Planococcus massiliensis* strain ES2 | 98.95 | MN577370.1 | Firmicutes |
| K86 | TV22 | Leaf | NA | *Janibacter* sp. | *Janibacter melonis* strain CM2104 | 99.5 | MN577371.1 | Actinobacteria |
| K87 | TV22 | Root | ISP7 | *Bacillus* sp. | *Bacillus licheniformis* strain DSM 13 | 99.93 | MN577372.1 | Firmicutes |
| K88 | TV22 | Root | ISP7 | *Bacillus* sp. | *Bacillus wiedmannii* strain FSL W8-0169 | 99.86 | MW905607 | Firmicutes |
| K89 | TV22 | Root | NA | *Bacillus* sp. | *Bacillus licheniformis* strain BCRC 11702 | 99.93 | MW898449 | Firmicutes |
| K91 | TV22 | Root | AIA | *Microbacterium* sp. | *Microbacterium azadirachtae* strain AI-S262 | 99.02 | MW898683 | Actinobacteria |
| K92 | TV22 | Root | AIA | *Microbacterium* sp. | *Microbacterium resistens* strain DMMZ 1710 | 98.32 | MW898684 | Actinobacteria |
| K96 | TV25 | Root | ISP7 | *Stenotrophomonas* sp. | *Stenotrophomonas maltophilia* strain ATCC 13637 | 98.64 | MW905624 | Proteobacteria |
| K97 | TV25 | Root | PKA | *Stenotrophomonas* sp. | *Stenotrophomonas maltophilia* strain ATCC 13637 | 99.79 | MN577375.1 | Proteobacteria |
| K98 | TV25 | Root | PKA | *Bacillus* sp. | *Bacillus safensis* strain NBRC 100820 | 99.79 | MN577376.1 | Firmicutes |
| K101 | TV25 | Leaf | PA | *Bacillus* sp. | *Bacillus aerius* strain 24K | 100 | MN577377.1 | Firmicutes |
| M2 | TV1 | Leaf | PKA | *Pseudomonas* sp. | *Pseudomonas koreensis* strain Ps 9-14 | 99.16 | MW905608 | Proteobacteria |
| M3 | TV1 | Leaf | SA | *Bacillus* sp. | *Bacillus subtilis* strain NBRC 13719 | 99.65 | MN577378.1 | Firmicutes |
| M5 | TV1 | Leaf | ISP7 | *Alcaligenes* sp. | *Alcaligenes faecalis* strain NBRC 13111 | 99.51 | MW905609 | Proteobacteria |
| M6 | TV1 | Leaf | KBA | *Alcaligenes* sp. | *Alcaligenes faecalis* strain NBRC 13111 | 99.51 | MW905610 | Proteobacteria |
| M9 | TV1 | Leaf | PKA | *Alcaligenes* sp. | *Alcaligenes faecalis* strain NBRC 13111 | 99.58 | MW905611 | Proteobacteria |
| M10 | TV1 | Leaf | NA | *Bacillus* sp. | *Bacillus velezensis* strain CBMB205 | 97.24 | MN577379.1 | Firmicutes |
| M11 | TV9 | Leaf | NA | *Brevibacillus* sp. | *Brevibacillus agrii* strain DSM 6348 | 99.93 | MN577380.1 | Firmicutes |
| M12 | TV22 | Leaf | NA | *Alcaligenes* sp. | *Alcaligenes faecalis* strain NBRC 13111 | 99.65 | MW905612 | Proteobacteria |
| M13 | TV22 | Leaf | PKA | *Paenibacillus* sp. | *Paenibacillus lautus* strain NBRC 15380 | 98.95 | MN577381.1 | Firmicutes |
| M14 | TV22 | Leaf | PKA | *Alcaligenes* sp. | *Alcaligenes faecalis* strain IAM 12369 | 99.58 | MW905613 | Proteobacteria |
| M15 | Teenali 17 | Leaf | KBA | *Microbacterium* sp. | *Microbacterium tumbae* strain T7528-3-6b | 98.17 | MW898685 | Actinobacteria |
| M17 | Teenali 17 | Leaf | SA | *Acinetobacter* sp. | *Acinetobacter rudis* strain G30 | 99.72 | MN577382.1 | Proteobacteria |
| M18 | Teenali 17 | Leaf | PKA | *Curtobacterium* sp. | *Curtobacterium oceanosedimentum* strain ATCC 31317 | 99.65 | MN577383.1 | Actinobacteria |
| M19 | Teenali 17 | Leaf | PKA | *Alcaligenes* sp. | *Alcaligenes faecalis* strain IAM 12369 | 99.65 | MW905614 | Proteobacteria |
| M21 | TV9 | Root | KBA | *Bacillus* sp. | *Bacillus haynesii* strain NRRL B-41327 | 99.65 | MW905615 | Firmicutes |
| M22 | TV9 | Root | PKA | *Bacillus* sp. | *Bacillus haynesii* strain NRRL B-41327 | 99.51 | MN577384.1 | Firmicutes |
| M23 | TV9 | Root | NA | *Bacillus* sp. | *Bacillus haynesii* strain NRRL B-41327 | 99.65 | MW905616 | Firmicutes |
| M24 | TV9 | Root | NA | *Bacillus* sp. | *Bacillus haynesii* strain NRRL B-41327 | 99.58 | MW905617 | Firmicutes |
| M25 | TV9 | Root | NA | *Stenotrophomonas* sp. | *Stenotrophomonas rhizophilia* strain e-p10 | 99.03 | MN577385.1 | Proteobacteria |
| M26 | TV9 | Root | NA | *Bacillus* sp. | *Bacillus haynesii* strain NRRL B-41327 | 99.65 | MW905618 | Firmicutes |
| M27 | TV9 | Root | PKA | *Alcaligenes* sp. | *Alcaligenes faecalis* strain IAM 12369 | 99.37 | MW905619 | Proteobacteria |
| M29 | TV1 | Root | KBA | *Pseudomonas* sp. | *Pseudomonas koreensis* strain Ps 9-14 | 99.3 | MW905620 | Proteobacteria |
| M30 | TV1 | Root | NA | *Pseudomonas* sp. | *Pseudomonas koreensis* strain Ps 9-14 | 99.23 | MW905621 | Proteobacteria |
| M34 | TV22 | Root | NA | *Microbacterium* sp. | *Microbacterium tumbae* strain T7528-3-6b | 98.17 | MW898686 | Actinobacteria |
| M35 | TV22 | Root | PKA | *Advenella* sp. | *Advenella kashmirensis* strain WT001 | 99.44 | MN577386.1 | Proteobacteria |
| M36 | TV22 | Root | ISP7 | *Microbacterium* sp. | *Microbacterium tumbae* strain T7528-3-6b | 98.1 | MW898687 | Actinobacteria |
| M37 | TV22 | Root | PKA | *Pseudomonas* sp. | *Pseudomonas koreensis* strain Ps 9-14 | 99.37 | MW905622 | Proteobacteria |
| M38 | TV22 | Root | ISP7 | *Pseudomonas* sp. | *Pseudomonas koreensis* strain Ps 9-14 | 99.37 | MW905623 | Proteobacteria |
| M43 | Teenali 17 | Root | NA | *Pseudomonas* sp. | *Pseudomonas umsongensis* strain Ps 3-10 | 98.66 | MN577388.1 | Proteobacteria |
| M44 | Teenali 17 | Root | SA | *Bacillus* sp. | *Bacillus subtilis* strain NBRC 13719 | 99.79 | MN577389.1 | Firmicutes |
| M45 | Teenali 17 | Root | PKA | *Pseudomonas* sp. | *Pseudomonas parafulva* strain NBRC 16636= DSM 17004 | 100 | MZ008002 | Proteobacteria |

**Supplementary Table 2.** *In vitro* plant growth promoting traits shown by endophytic bacterial isolates

| Isolate | IAA production (µg mL^-1^) * | P solubilization (mg L^-1^) * | Ammonia production (µmol mL^-1^) * | Siderophore production (%) * | Nitrogen fixation | ACC deaminase activity | Cellulase activity | Protease activity | Amylase activity | Total assessment Points (13) | Rank |
| --- | --- | --- | --- | --- | --- | --- | --- | --- | --- | --- | --- |
| K1 | 7.42 ± 1.49 (1) | 0 (0) | 2.13 ± 0.25 (1) | 0 (0) | + (1) | + (1) | - (0) | + (1) | - (0) | 5 | 49^th^ |
| K2 | 3.00 ± 1.20 (1) | 0 (0) | 4.71 ± 0.37 (1) | 28.86 ± 3.5 (1) | + (1) | + (1) | - (0) | + (1) | - (0) | 6 | 20^th^ |
| K4 | 25.17 ± 0.82 (1) | 0 (0) | 4.43 ± 0.50 (1) | 83.22 ± 0.67(1) | - (0) | + (1) | + (1) | + (1) | - (0) | 6 | 21^st^ |
| K7 | 13.65 ± 0.12 (1) | 0 (0) | 2.29 ± 0.27 (1) | 0 (0) | + (1) | + (1) | - (0) | + (1) | + (1) | 6 | 22^nd^ |
| K8 | 18.29 ± 0.60 (1) | 0 (0) | 1.32 ± 0.27 (1) | 52.88 ± 0.42 (1) | - (0) | + (1) | + (1) | + (1) | - (0) | 6 | 23^rd^ |
| K9 | 5.06 ± 2.4 (1) | 0 (0) | 3.58 ± 0.14 (1) | 11.41 ± 1.5 (1) | + (1) | + (1) | + (1) | + (1) | + (1) | 8 | 6^th^ |
| K10 | 13.94 ± 1.05 (1) | 18.76 ± 3.28 (1) | 4.08 ± 0.18 (1) | 0 (0) | - (0) | + (1) | + (0) | + (1) | - (0) | 5 | 50^th^ |
| K11 | 16.09 ± 1.20 (1) | 0 (0) | 2.76 ± 0.29 (1) | 0 (0) | - (0) | + (1) | + (1) | - (0) | - (0) | 4 | 70^th^ |
| K12 | 7.14 ± 0.07 (1) | 0 (0) | 4.31 ± 0.62 (1) | 0 (0) | + (1) | + (1) | + (1) | + (1) | + (1) | 7 | 12^th^ |
| K13 | 10.81 ± 1.10 (1) | 0 (0) | 1.29 ± 0.40 (1) | 31.54 ± 0.78 (1) | - (0) | + (1) | - (0) | - (0) | - (0) | 4 | 71^st^ |
| K14 | 20.99 ± 0.94 (1) | 0 (0) | 2.58 ± 0.03 (1) | 0 (0) | - (0) | + (1) | - (0) | - (0) | + (1) | 4 | 72^nd^ |
| K15 | 0 (0) | 0 (0) | 0 (0) | 0 (0) | - (0) | + (1) | - (0) | - (0) | - (0) | 1 | 106^th^ |
| K16 | 26.62 ± 1.50 (1) | 0 (0) | 1.26 ± 0.51 (1) | 39.60 ± 0.24 (1) | + (1) | - (0) | - (0) | + (1) | - (0) | 5 | 51^st^ |
| K17 | 4.71 ± 0.94 (1) | 0 (0) | 1.02 ± 0.22 (1) | 0 (0) | + (1) | + (1) | - (0) | - (0) | + (1) | 5 | 52^nd^ |
| K18 | 34.53 ± 1.87 (1) | 26.42 ± 1.54 (1) | 4.74 ± 0.32 (1) | 0 (0) | + (1) | + (1) | - (0) | + (1) | - (0) | 6 | 24^th^ |
| K19 | 0 (0) | 11.70 ± 1.80 (1) | 1.41 ± 0.11 (1) | 0 (0) | - (0) | + (1) | - (0) | - (0) | - (0) | 3 | 99^th^ |
| K20 | 3.82 ± 1.39 (1) | 0 (0) | 1.82 ± 0.17 (1) | 0 (0) | + (1) | - (0) | - (0) | + (1) | - (0) | 4 | 73^rd^ |
| K21 | 18.68 ± 1.49 (1) | 0 (0) | 2.77 ± 0.13 (1) | 0 (0) | + (1) | + (1) | - (0) | - (0) | - (0) | 4 | 74^th^ |
| K22 | 7.14± 0.73 (1) | 0 (0) | 4.69 ± 0.20 (1) | 0 (0) | + (1) | + (1) | - (0) | - (0) | - (0) | 4 | 75th |
| K25 | 18.67 ± 1.21(1) | 29.86 ± 6.9 (1) | 4.68 ± 0.17 (1) | 0 (0) | + (1) | + (1) | - (0) | - (0) | - (0) | 5 | 53^rd^ |
| K26 | 18.98 ± 1.00 (1) | 50.38 ± 2.20 (2) | 4.57 ± 0.39 (1) | 45.90 ± 0.05 (1) | + (1) | - (0) | - (0) | - (0) | + (1) | 7 | 13^th^ |
| K27 | 26.48 ± 1.22 (1) | 0 (0) | 3.00 ± 0.14 (1) | 0 (0) | + (1) | + (1) | + (1) | - (0) | - (0) | 5 | 54^th^ |
| K30 | 20.59 ± 0.69 (1) | 12.56 ± 0.73 (1) | 1.90 ± 0.23 (1) | 0 (0) | + (1) | + (1) | - (0) | + (1) | - (0) | 6 | 25^th^ |
| K31 | 18.18 ± 2.04 (1) | 18.38 ± 1.80 (1) | 1.91 ± 0.23 (1) | 0 (0) | + (1) | + (1) | - (0) | + (1) | - (0) | 6 | 26^th^ |
| K33 | 0 (0) | 0 (0) | 0.61 ± 0.26 (1) | 0 (0) | + (1) | - (0) | + (1) | + (1) | - (0) | 4 | 76^th^ |
| K34 | 0 (0) | 0 (0) | 0.85 ± 0.26 (1) | 0 (0) | + (1) | - (0) | - (0) | + (1) | + (1) | 4 | 77^th^ |
| K36 | 23.92 ± 1.37 (1) | 26.77 ± 1.81 (1) | 1.61 ± 0.19 (1) | 0 (0) | + (1) | + (1) | - (0) | + (1) | - (0) | 6 | 27^th^ |
| K37 | 29.80 ± 1.30 (1) | 0 (0) | 1.94 ± 0.30 (1) | 0 (0) | + (1) | - (0) | + (1) | - (0) | - (0) | 4 | 78^th^ |
| K38 | 30.60 ± 1.42 (1) | 0 (0) | 2.78 ± 0.31 (1) | 0 (0) | + (1) | - (0) | - (0) | - (0) | - (0) | 3 | 100^th^ |
| K39 | 129.84 ± 4.73 (3) | 0 (0) | 2.15 ± 0.11 (1) | 0 (0) | + (1) | - (0) | - (0) | - (0) | - (0) | 5 | 55^th^ |
| K41 | 4.00 ± 1.32 (1) | 0 (0) | 0.96 ± 0.21 (1) | 0 (0) | + (1) | + (1) | - (0) | - (0) | - (0) | 4 | 79^th^ |
| K42 | 9.87 ± 1.10 (1) | 0 (0) | 1.99 ± 0.24 (1) | 0 (0) | + (1) | + (1) | - (0) | + (1) | + (1) | 6 | 28^th^ |
| K44 | 21.20 ± 0.81 (1) | 0 (0) | 2.74 ± 0.03 (1) | 0 (0) | + (1) | + (1) | - (0) | + (1) | - (0) | 5 | 56^th^ |
| K45 | 22.36 ± 1.50 (1) | 0 (0) | 2.28 ± 0.16 (1) | 0 (0) | + (1) | + (1) | - (0) | + (1) | - (0) | 5 | 57^th^ |
| K46 | 19.59 ± 7.94 (1) | 0 (0) | 1.63 ± 0.33 (1) | 0 (0) | + (1) | + (1) | - (0) | - (0) | - (0) | 4 | 80^th^ |
| K49 | 0 (0) | 0 (0) | 3.34 ± 0.30 (1) | 0 (0) | + (1) | + (1) | - (0) | + (1) | - (0) | 4 | 81^st^ |
| K50 | 0 (0) | 0 (0) | 1.29 ± 0.26 (1) | 0 (0) | + (1) | + (1) | + (1) | - (0) | + (1) | 5 | 58^th^ |
| K51 | 37.68 ± 0.63 (1) | 0 (0) | 1.91 ± 0.08 (1) | 8.724 ± 0.06 (1) | + (1) | + (1) | + (1) | + (1) | + (1) | 8 | 7^th^ |
| K53 | 9.08 ± 2.51 (1) | 0 (0) | 4.76 ± 0.20 (1) | 32.35 ± 0.26 (1) | + (1) | + (1) | - (0) | + (1) | - (0) | 6 | 29^th^ |
| K54 | 7.68 ± 2.41 (1) | 0 (0) | 3.51 ± 0.72 (1) | 0 (0) | + (1) | + (1) | - (0) | - (0) | - (0) | 4 | 82^nd^ |
| K55 | 68.58 ± 1.01 (2) | 7.11 ± 1.61 (1) | 1.61 ± 0.16 (1) | 0 (0) | + (1) | + (1) | + (1) | + (1) | + (1) | 9 | 3^rd^ |
| K56 | 6.23 ± 1.07 (1) | 0 (0) | 4.74 ± 0.33 (1) | 0 (0) | + (1) | + (1) | - (0) | + (1) | + (1) | 6 | 30^th^ |
| K57 | 31.56 ± 1.00 (1) | 0 (0) | 2.61 ± 0.14 (1) | 0 (0) | + (1) | + (1) | + (1) | + (1) | - (0) | 6 | 31^st^ |
| K58 | 3.86 ± 0.99 (1) | 24.89 ± 0.57 (1) | 1.73 ± 0.27 (1) | 0 (0) | + (1) | + (1) | - (0) | - (0) | + (1) | 6 | 32^nd^ |
| K59 | 3.30 ± 2.23 (1) | 36.33 ± 0.86 (2) | 2.44 ± 0.14 (1) | 0 (0) | - (0) | + (1) | - (0) | - (0) | - (0) | 5 | 59^th^ |
| K61 | 25.62 ± 1.18 (1) | 73.24 ± 0.39 (3) | 2.15 ± 0.23 (1) | 0 (0) | + (1) | + (1) | + (1) | + (1) | - (0) | 9 | 4^th^ |
| K63 | 4.72 ± 1.19 (1) | 2.79 ± 0.68 (1) | 1.91 ± 0.39 (1) | 52.08 ± 0.06 (1) | + (1) | + (1) | - (0) | + (1) | - (0) | 7 | 14^th^ |
| K64 | 3.01 ± 1.09 (1) | 0 (0) | 2.47 ± 0.18 (1) | 71.41 ± 0.09 (1) | + (1) | + (1) | - (0) | - (0) | + (1) | 6 | 33^rd^ |
| K66 | 0 (0) | 0 (0) | 1.96 ± 0.20 (1) | 27.52 ± 0.35 (1) | + (1) | + (1) | - (0) | - (0) | - (0) | 4 | 83^rd^ |
| K67 | 27.45 ± 1.26 (1) | 0 (0) | 2.70 ± 0.16 (1) | 0 (0) | + (1) | + (1) | - (0) | - (0) | + (1) | 5 | 60^th^ |
| K68 | 0 (0) | 0 (0) | 0.78 ± 0.17 (1) | 21.20 ± 0.06 (1) | - (0) | + (1) | - (0) | - (0) | + (1) | 4 | 84^th^ |
| K69 | 22.20 ± 1.16 (1) | 53.92 ± 8.80 (2) | 3.69 ± 0.26 (1) | 0 (0) | - (0) | + (1) | - (0) | - (0) | + (1) | 6 | 34^th^ |
| K70 | 4.07 ± 0.54 (1) | 51.60 ± 3.90 (2) | 0 (0) | 0 (0) | + (1) | + (1) | + (1) | - (0) | - (0) | 6 | 35^th^ |
| K71 | 32.25 ± 1.44 (1) | 51.02 ± 1.26 (2) | 0.66 ± 0.15 (1) | 67.65 ± 0.16 (1) | + (1) | + (1) | - (0) | + (1) | - (0) | 8 | 8^th^ |
| K72 | 0 (0) | 0 (0) | 0 (0) | 0 (0) | + (1) | + (1) | - (0) | + (1) | - (0) | 3 | 101^st^ |
| K73 | 0 (0) | 0 (0) | 2.63 ± 0.22 (1) | 58.66 ± 0.76 (1) | + (1) | + (1) | + (1) | + (1) | - (0) | 6 | 36^th^ |
| K74 | 0 (0) | 0 (0) | 2.67 ± 0.40 (1) | 0 (0) | + (1) | + (1) | - (0) | + (1) | - (0) | 4 | 85^th^ |
| K75 | 8.75 ± 1.20 (1) | 0 (0) | 2.38 ± 0.19 (1) | 0 (0) | + (1) | + (1) | + (1) | + (1) | - (0) | 6 | 37^th^ |
| K76 | 0 (0) | 0 (0) | 2.17 ± 0.19 (1) | 53.15 ± 1.3 (1) | + (1) | + (1) | + (1) | - (0) | - (0) | 5 | 61^st^ |
| K78 | 16.05 ± 1.04 (1) | 0 (0) | 1.48 ± 0.13 (1) | 0 (0) | + (1) | + (1) | - (0) | - (0) | - (0) | 4 | 86^th^ |
| K80 | 29.58 ± 1.33 (1) | 0 (0) | 1.51 ± 0.07 (1) | 35.57 ± 2.4 (1) | + (1) | + (1) | + (1) | - (0) | - (0) | 6 | 38^th^ |
| K81 | 36.35 ± 1.19 (1) | 0 (0) | 1.35 ± 0.31 (1) | 0 (0) | + (1) | + (1) | + (1) | - (0) | - (0) | 5 | 62^nd^ |
| K82 | 43.12 ± 0.93 (1) | 11.33 ± 1.81 (1) | 1.21 ± 0.21 (1) | 0 (0) | + (1) | + (1) | - (0) | + (1) | - (0) | 6 | 39^th^ |
| K84 | 11.93 ± 0.60 (1) | 0 (0) | 3.84 ± 0.12 (1) | 0 (0) | + (1) | + (1) | - (0) | + (1) | - (0) | 5 | 63^rd^ |
| K85 | 0 (0) | 49.37 ± 3.96 (2) | 0 (0) | 44.29 ± 0.87 (1) | + (1) | + (1) | - (0) | + (1) | - (0) | 6 | 40^th^ |
| K86 | 23.79 ± 1.60 (1) | 0 (0) | 1.50 ± 0.30 (1) | 67.11 ± 0.09 (1) | + (1) | + (1) | - (0) | + (1) | - (0) | 6 | 41^st^ |
| K87 | 5.58 ± 1.34 (1) | 0 (0) | 4.50 ± 0.23 (1) | 0 (0) | + (1) | + (1) | - (0) | - (0) | - (0) | 4 | 87^th^ |
| K88 | 19.52 ± 0.54 (1) | 0 (0) | 4.37 ± 0.09 (1) | 0 (0) | + (1) | + (1) | + (1) | + (1) | - (0) | 6 | 42^nd^ |
| K89 | 11.11 ± 0.51(1) | 0 (0) | 4.74 ± 0.22 (1) | 0 (0) | + (1) | + (1) | + (1) | + (1) | - (0) | 6 | 43^rd^ |
| K91 | 11.14 ± 8.85 (1) | 0 (0) | 4.50 ± 0.19 (1) | 14.09 ± 0.52 (1) | - (0) | + (1) | + (1) | + (1) | - (0) | 6 | 44^th^ |
| K92 | 5.69 ± 1.84 (1) | 0 (0) | 2.63 ± 0.20 (1) | 24.43 ± 0.85 (1) | - (0) | + (1) | - (0) | - (0) | - (0) | 4 | 88^th^ |
| K96 | 123.56 ± 2.48 (3) | 71.63 ± 2.20 (3) | 4.61 ± 0.54 (1) | 79.06 ± 0.45 (1) | + (1) | + (1) | + (1) | + (1) | + (1) | 13 | 1^st^ |
| K97 | 82.32 ± 5.13 (2) | 0 (0) | 1.11 ± 0.33 (1) | 83.22 ± 0.68 (1) | + (1) | + (1) | - (0) | + (1) | - (0) | 7 | 15^th^ |
| K98 | 0 (0) | 81.42 ± 5.73 (3) | 1.44 ± 0.07 (1) | 41.47 ± 1.6 (1) | + (1) | + (1) | - (0) | + (1) | + (1) | 9 | 5^th^ |
| K101 | 5.16 ± 0.70 (1) | 0 (0) | 0 (0) | 17.72 ± 1.8 (1) | + (1) | + (1) | - (0) | + (1) | - (0) | 5 | 64^th^ |
| M2 | 11.77 ± 0.52 (1) | 29.93 ± 1.61 (1) | 3.03 ± 0.15 (1) | 42.3 ± 0.01 (1) | + (1) | + (1) | + (1) | + (1) | - (0) | 8 | 9^th^ |
| M3 | 12.15 ± 3.20 (1) | 67.00 ± 6.26 (3) | 4.64 ± 0.20 (1) | 0 (0) | + (1) | + (1) | - (0) | + (1) | - (0) | 8 | 10^th^ |
| M5 | 54.14 ± 10.60 (2) | 0 (0) | 1.49 ± 0.14 (1) | 0 (0) | - (0) | + (1) | - (0) | - (0) | - (0) | 4 | 89^th^ |
| M6 | 44.70 ± 0.32 (1) | 0 (0) | 1.34 ± 0.13 (1) | 0 (0) | + (1) | + (1) | - (0) | - (0) | - (0) | 4 | 90^th^ |
| M9 | 20.90 ± 4.47 (1) | 0 (0) | 1.05 ± 0.15 (1) | 20.8 ± 1.1 (1) | + (1) | - (0) | - (0) | - (0) | - (0) | 4 | 91^st^ |
| M10 | 20.83 ± 0.80 (1) | 0 (0) | 4.65 ± 0.36 (1) | 6.17 ± 0.64 (1) | + (1) | + (1) | + (1) | + (1) | - (0) | 7 | 16^th^ |
| M11 | 96.31 ± 10.80 (2) | 0 (0) | 2.01 ± 0.19 (1) | 0 (0) | - (0) | + (1) | - (0) | - (0) | - (0) | 4 | 92^nd^ |
| M12 | 15.90 ± 0.60 (1) | 55.10 ± 2.80 (2) | 1.68 ± 0.23 (1) | 0 (0) | - (0) | + (1) | - (0) | + (1) | - (0) | 6 | 45^th^ |
| M13 | 42.16 ± 2.00 (1) | 0 (0) | 1.90 ± 0.37 (1) | 0 (0) | + (1) | - (0) | - (0) | - (0) | - (0) | 3 | 102^nd^ |
| M14 | 12.51 ± 5.2 (1) | 0 (0) | 2.16 ± 0.70 (1) | 0 (0) | - (0) | - (0) | - (0) | + (1) | - (0) | 3 | 103^rd^ |
| M15 | 14.26 ± 1.40 (1) | 0 (0) | 4.92 ± 0.19 (1) | 0 (0) | - (0) | + (1) | + (1) | - (0) | - (0) | 4 | 93^rd^ |
| M17 | 10.50 ± 2.23 (1) | 0 (0) | 3.74 ± 0.49 (1) | 0 (0) | - (0) | + (1) | + (1) | - (0) | - (0) | 4 | 94^th^ |
| M18 | 21.35 ± 2.17 (1) | 27.36 ± 1.19 (1) | 2.54 ± 0.41 (1) | 0 (0) | - (0) | + (1) | - (0) | + (1) | - (0) | 5 | 65^th^ |
| M19 | 25.55 ±0.51 (1) | 0 (0) | 4.80 ± 0.40 (1) | 0 (0) | + (1) | + (1) | + (1) | - (0) | - (0) | 5 | 66^th^ |
| M21 | 3.67 ± 0.44 (1) | 0 (0) | 4.26 ± 0.45 (1) | 0 (0) | + (1) | + (1) | - (0) | - (0) | - (0) | 4 | 95^th^ |
| M22 | 4.35 ± 0.62 (1) | 0 (0) | 4.92 ± 0.19 (1) | 0 (0) | + (1) | + (1) | - (0) | - (0) | - (0) | 4 | 96^th^ |
| M23 | 3.10 ± 0.55 (1) | 0 (0) | 4.73 ± 0.34 (1) | 19.20 ± 0.74 (1) | + (1) | + (1) | - (0) | - (0) | - (0) | 5 | 67^th^ |
| M24 | 7.60 ± 1.23 (1) | 0 (0) | 3.47 ± 0.28 (1) | 0 (0) | + (1) | - (0) | - (0) | + (1) | - (0) | 4 | 97^th^ |
| M25 | 8.88 ± 0.61(1) | 0 (0) | 4.49 ± 0.20 (1) | 0 (0) | + (1) | + (1) | + (1) | + (1) | - (0) | 6 | 46^th^ |
| M26 | 5.71 ± 1.22 (1) | 0 (0) | 4.10 ± 0.14 (1) | 0 (0) | + (1) | + (1) | + (1) | - (0) | - (0) | 5 | 68^th^ |
| M27 | 11.58 ± 2.37 (1) | 0 (0) | 4.16 ± 0.17 (1) | 0 (0) | - (0) | + (1) | - (0) | - (0) | - (0) | 3 | 104^th^ |
| M29 | 79.84 ± 1.85 (2) | 27.18 ± 6.01(1) | 2.49 ± 0.12 (1) | 5.37 ± 0.023 (1) | + (1) | + (1) | + (1) | - (0) | - (0) | 8 | 11^th^ |
| M30 | 14.77 ± 1.00 (1) | 0 (0) | 2.15 ± 0.07 (1) | 0 (0) | + (1) | - (0) | - (0) | - (0) | - (0) | 3 | 105^th^ |
| M34 | 8.10 ± 1.10 (1) | 0 (0) | 4.32 ± 0.31 (1) | 0 (0) | + (1) | + (1) | + (1) | - (0) | - (0) | 5 | 69^th^ |
| M35 | 70.37 ± 1.50 (2) | 71.54 ± 3.03 (3) | 0.77 ± 0.35 (1) | 0 (0) | - (0) | + (1) | - (0) | - (0) | - (0) | 7 | 17^th^ |
| M36 | 3.09 ± 1.20 (1) | 0 (0) | 2.65 ± 0.18 (1) | 0 (0) | - (0) | + (1) | + (1) | - (0) | - (0) | 4 | 98^th^ |
| M37 | 36.80 ± 2.60 (1) | 19.09 ± 3.93 (1) | 4.18 ± 0.32 (1) | 40.27 ± 1.7 (1) | + (1) | + (1) | - (0) | + (1) | - (0) | 7 | 18^th^ |
| M38 | 14.81 ± 1.64 (1) | 0 (0) | 1.50 ± 0.34 (1) | 0 (0) | + (1) | + (1) | + (1) | + (1) | - (0) | 6 | 47^th^ |
| M43 | 21.72 ± 5.43 (1) | 12.63 ± 1.21 (1) | 1.56 ± 0.27 (1) | 0 (0) | + (1) | + (1) | - (0) | + (1) | - (0) | 6 | 48^th^ |
| M44 | 6.70 ± 1.50 (1) | 17.28 ± 1.21 (1) | 4.92 ± 0.19 (1) | ­- (0) | + (1) | + (1) | ­- (0) | + (1) | + (1) | 7 | 19^th^ |
| M45 | 88.00 ± 0.50 (2) | 58.16 ± 2.34 (2) | 4.31 ± 0.06 (1) | 81.20 ± 1.1 (1) | + (1) | + (1) | + (1) | + (1) | + (1) | 11 | 2^nd^ |

*The values represent mean value (n=3), ± (standard deviation, SE), + activity or growth, - represents negative activity or no growth. The numbers in parentheses indicates the bonitur assessment score.0 indicates no activity or growth. IAA^a^ - Indole Acetic Acid production (1 = < 50, 2 = 50 - 100, 3 = > 100 µg mL^-1^); PS^b^ - Phosphate solubilization (1 = < 30, 2 = 30 - 60, 3 = > 60 µg mL^-1^); Amm^c^ - Ammonia production (µmol mL^-1^) (1= positive); Sid^d^ -Siderophore production (%) (1 = positive); Nit^e^ - Nitrogen fixation (1= positive); ACC^f^ - ACC deaminase activity (1= positive); Cel^g^ - Cellulase activity (1= positive); Pro^h^ - Protease activity (1=positive); Amy^i^ - Amylase activity (1=positive). 0 indicates no activity or growth.
